# Simultaneous capture of single cell RNA-seq, ATAC-seq, and CRISPR perturbation enables multiomic screens to identify gene regulatory relationships

**DOI:** 10.1016/j.crmeth.2025.101222

**Published:** 2025-11-10

**Authors:** Kaivalya Shevade, Yeqing Angela Yang, Kevin Feng, Karl Mader, Volkan Sevim, Jacob Parsons, Gunisha Arora, Hasnaa Elfawy, Rachel Mace, Scot Federman, Rustam Esanov, Shawn Shafer, Eric D. Chow, Laralynne Przybyla

**Affiliations:** 1 Laboratory for Genomics Research, San Francisco, CA 94158, USA; 2 Department of Biochemistry and Biophysics, University of California, San Francisco, CA 94158, USA; 3 GSK, San Francisco, CA, USA; 4 GSK, Collegeville, PA, USA; 5 Center for Advanced Technology, University of California, San Francisco, CA 94158, USA

**Keywords:** multiome, single cell, CRISPR, guide RNA, perturb-seq

## Abstract

Here, we introduce CRISPR and transcriptomics-assay for transposase-accessible chromatin (CAT-ATAC), a technique that adds CRISPR guide RNA (gRNA) capture to the existing 10× Genomics Multiome assay, generating linked transcriptome, chromatin accessibility, and perturbation identity data from the same individual cells. We demonstrate up to 77% capture rate for both arrayed and pooled delivery of lentiviral gRNAs in induced pluripotent stem cells (iPSCs) and cancer cell lines. This capability allows us to construct gene regulatory networks (GRNs) in cells under drug and genetic perturbations. By applying CAT-ATAC, we identified a GRN associated with dasatinib resistance, indirectly activated by the HIC2 gene. Using loss-of-function experiments, we further validated that ZFPM2, a component of the predicted GRN, also contributes to dasatinib resistance. CAT-ATAC can thus be used to generate high-content multidimensional genotype-phenotype maps to reveal gene and cellular interactions and functions.

## Highlights

• Assay linking CRISPR perturbation, gene expression, and chromatin accessibility
• Easy to implement using commercially available 10× Genomics Multiome assay
• Gene expression and chromatin data quality equivalent to unmodified assay
• Can be used to construct gene regulatory networks associated with drug resistance

## Motivation

Single-cell functional genomics methods, such as Perturb-seq and Spear-ATAC, have made it possible to achieve high-throughput mapping of the functional effects of gene perturbations by profiling transcriptomes and DNA accessibility. Combining single-cell chromatin accessibility and transcriptomic data via multiomic approaches has facilitated the discovery of novel *cis* and gene regulatory interactions. However, pseudobulk readouts from cell populations can often cloud the interpretation of results due to a heterogeneous response from cells receiving the same genetic perturbation, which could be mitigated by using transcriptional profiles of single cells to subset the ATAC-seq data. Existing methods to capture CRISPR guide RNAs to simultaneously assess the impact of genetic perturbations on RNA and ATAC profiles require either cloning gRNA libraries in specialized vectors or implementing complex protocols with multiple rounds of barcoding. We sought to develop a method to enable CRISPR guide capture in the existing 10× Genomics workflow, generating linked transcriptome, chromatin accessibility, and perturbation identity data from the same individual cells.

## Graphical abstract

**Figure:**
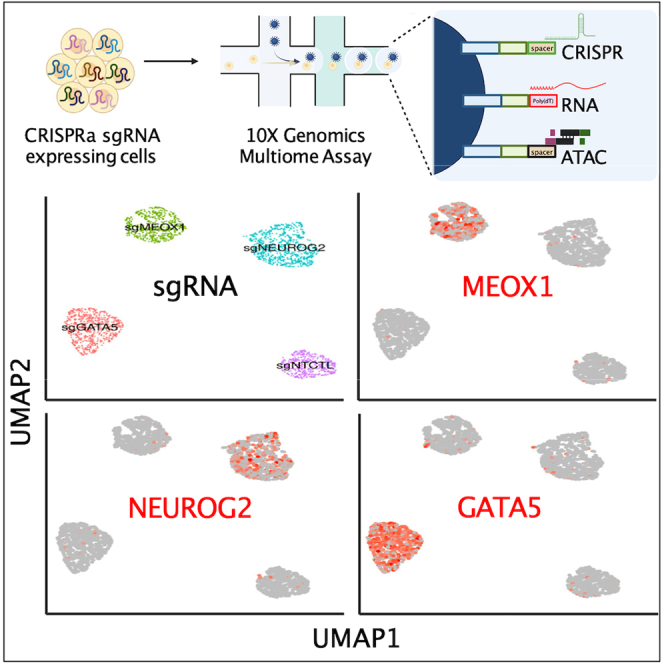

## Teaser

Shevade et al. present CAT-ATAC (CRISPR and transcriptomics-assay for transposase-accessible chromatin), a single-cell sequencing assay that profiles gene expression, chromatin accessibility, and CRISPR perturbation from the same cells by modifying the commercially available 10× Multiome assay, and demonstrate its utility in multiple biological and therapeutically relevant applications.

## Introduction

Since the discovery of CRISPR-Cas9 genome editing over a decade ago, researchers have been adopting the system in a wide range of applications due to its high efficiency and ability to target specific genomic loci. CRISPR-mediated perturbations (knockout, interference, and activation) provide a powerful tool to interrogate gene functions in a systematic manner. Given its ease of programmability, CRISPR-Cas9 can be combined with genome-wide guide RNA (gRNA) libraries for conducting unbiased and high-throughput genetic screens.^1^ Different phenotypic assays can be utilized to evaluate the outcome of a pooled CRISPR screen, including gRNA representation analysis and single-cell evaluation.^2^^,^^3^^,^^4^ By using single-cell RNA sequencing (scRNA-seq) as a readout for pooled CRISPR screens, methods such as Perturb-seq,^5^^,^^6^ CRISP-seq,^7^ and CROP-seq^8^ allow us to dive deep into the relationship between every genetic perturbation and its corresponding transcriptomic outcomes. These functional genomic studies provide information-rich phenotypic profiling that can significantly accelerate our understanding of biological and pathological processes.

In addition to transcriptomic changes, epigenomic effects are often as informative and crucial to understanding transcriptional regulation in a complex system. The dynamics of chromatin accessibility, dictated by transcription factor occupancy and epigenetic mechanisms such as histone modifications and DNA methylation, underlie many fundamental biological processes in development and disease.^9^^,^^10^ Thus, single-cell ATAC-seq (assay for transposase-accessible chromatin with sequencing),^11^ which measures chromatin accessibility in individual cells, has been implemented as a readout for pooled CRISPR screens in methods such as Perturb-ATAC,^12^ CRISPR-sciATAC,^13^ and Spear-ATAC.^14^ These methods employ various single-cell platforms and have guide capture efficiencies ranging from 40% to 90%. As an example of their utility, recently published atlases detailing single-cell chromatin accessibility in various tissues offer abundant insights into cell type-specific *cis*-regulatory elements and how they can be used to predict links between non-coding disease risk variants to downstream affected genes and functions.^15^^,^^16^ Single-cell profiling techniques such as these can provide novel insights into gene regulatory networks (GRNs) that drive several complex biological processes, with applications to developmental trajectories and disease mechanisms.

Drug resistance to chemotherapeutics and/or targeted therapies is a known problem in cancer and can account for almost 90% of cancer-associated deaths.^17^ Chronic myeloid leukemia (CML) develops from a recombination between chromosomes 9 and 22, creating a short chromosome 22 (Philadelphia chromosome) in myeloid cells that results in a BCR-ABL gene fusion, causing constitutive activation of the Abl tyrosine kinase. Dasatinib, a front-line treatment for CML, is a broad-spectrum tyrosine kinase inhibitor (TKI)^18^ like most other CML treatments. The development of TKIs has increased the 5-year survival rate from 22% in the mid 1970s to 69%.^19^ However, there is no complete cure for the disease. Although dasatinib and other TKIs effectively block the Abl kinase, drug resistance ultimately leads to treatment failure. In as many as 40% of cases of clinical TKI failure, cancer cells exhibit sustained BCR-ABL inhibition^20^ but also activate alternative pathways that help them survive in the presence of the drug. Identification of BCR-ABL-independent mechanisms of drug resistance could thus lead to new therapeutic targets for CML.^21^^,^^22^ Understanding the dynamics of transcriptome and DNA accessibility in response to genetic and drug perturbation might help us piece together the novel gene regulatory relationships that underlie drug resistance by providing a deeper understanding of the direct and indirect effects of alternative pathway activation.

To interrogate both the transcriptional profile and the epigenetic landscape in CRISPR screens, we have developed CRISPR and transcriptomics-assay for transposase-accessible chromatin (CAT-ATAC). This method simultaneously profiles the transcriptome, chromatin accessibility, and CRISPR gRNA expression within single cells by leveraging the Multiome assay kit from 10× Genomics and is compatible with the widely used Perturb-seq dual-guide vector backbone and is easily customized for other gRNA vectors. The pairing of the two layers of data, transcriptional and regulatory states, at single-cell resolution provides a multidimensional phenotypic readout that will enable CRISPR-based screens in a range of applications intended to uncover gene-regulatory networks. In this study, we developed CAT-ATAC in induced pluripotent stem cells (iPSCs) and used it to elucidate a gene regulatory network that underlies dasatinib drug resistance in CML cells.

## Results

### CAT-ATAC methodology and proof-of-concept

**Figure 1:**
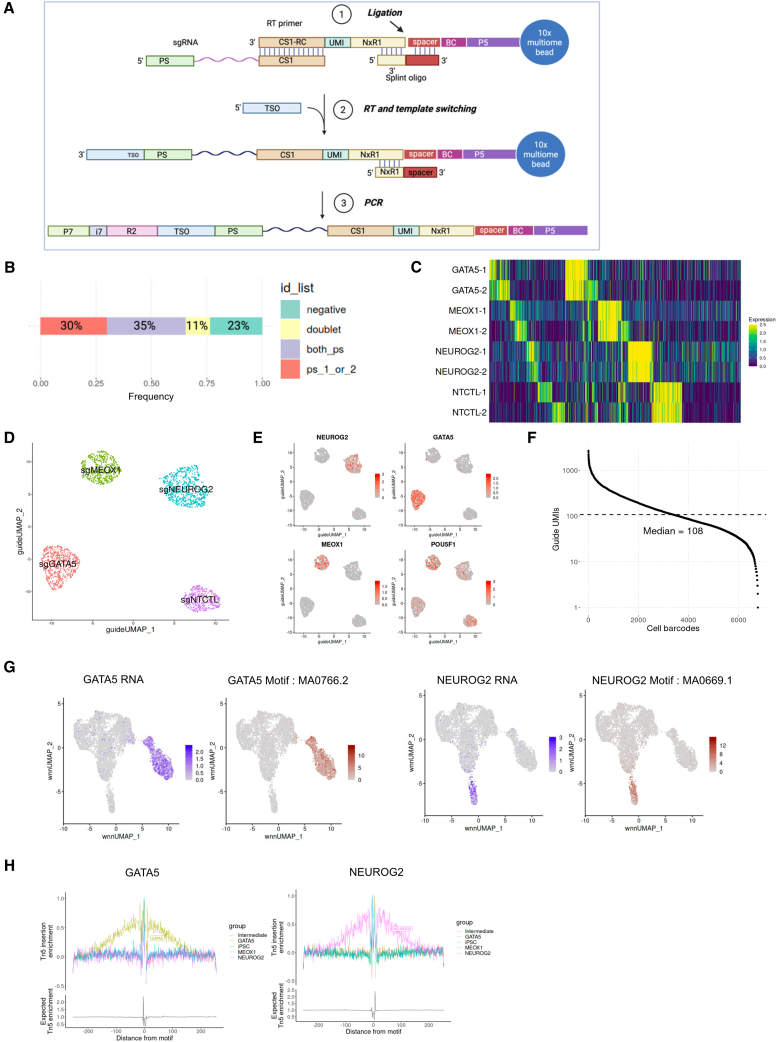
CAT-ATAC method and validation (A) Detailed schematic illustrating the guide capture process. (B) Overall guide assignment for the pilot experiment; 76% of the total cells could be assigned to guides, 11% of which were identified as doublets. (C) Heatmap showing guide assignment in all 6,656 cells. (D) UMAP embedding of singlets expressing 2 protospacers (Singlet_2_PS) based on guide expression. (E) RNA expression plots of targeted genes in guide expression UMAP. (F) Barcode rank plot for sgRNA UMI counts, showing a median of 108 UMIs for captured guide sequences per cell. (G) Expression of the TF shown in purple, motif enrichment shown in brown. (H) TF footprinting for GATA5 and NEUROG2.

CAT-ATAC is built on the 10× Genomics Multiome assay, which uses a droplet-based method to capture both RNA and chromatin fragments. In the workflow (Figure S1A), dual-gRNA constructs^23^ are introduced into cells that express Cas9-CRISPR machinery (e.g., CRISPRn, CRISPRi, or CRISPRa). CAT-ATAC reagents are combined with tagmented nuclei and passed through the 10× Genomics single-cell controller to compartmentalize each nucleus into an oil droplet containing a mix of reagents, enzymes and oligo primers. Through downstream thermocycling reactions and enrichment steps, the mRNA transcripts, ATAC fragments, and the gRNA transcripts within each cell are barcoded with a unique sequence and constructed into three libraries, one each for assaying RNA, ATAC, and gRNA. Figure 1A details the steps involved in capturing gRNAs, which involves adding a pre-annealed reverse transcription (RT) primer with a splint oligo to reverse transcribe the gRNA. The splint oligo is complementary to the spacer sequence on the ATAC-capture oligo of the 10× Multiome gel beads and the gRNA RT products. The RT primer contains a sequence complementary to the capture sequence (CS1) found in gRNAs expressed from the dual-guide Perturb-seq gRNA construct,^24^ a unique molecular identifier (UMI), and the Nextera R1 sequence. This primer forms a duplex with the splint oligo through a partial Nextera R1 sequence. During RT and template switching, the spiked-in primer initiates first strand cDNA synthesis of the gRNA followed by template switching. Subsequent rounds of PCR amplification add appropriate adaptors and sample indexes to make the final single-cell gRNA library. Through optimization experiments, we found that some changes to the 10× Multiome GEM incubation step were needed for better performance of capturing all three modalities: mRNA, ATAC, and gRNA simultaneously (data not shown). We increased the RT temperature from 37^o^C to 53^o^C to enhance RT efficiency of gRNAs that have significant secondary structures such as hairpins. In standard Perturb-seq experiments, RT typically happens at 53^o^C. However, we noticed that when we simply increased RT to 53^o^C followed by ligation, we were not able to generate the ATAC library. This is hypothesized to result from the overhangs of tagmented DNA being filled by the more active RT enzyme at this higher temperature and thus not allowing ATAC fragments to be ligated to gel bead oligos. Therefore, we avoided this problem by including a ligation step before RT to join the ATAC fragments onto gel bead oligos, and then carry out RT at the increased temperature. We used Hi-T4 ligase to ensure that the ligase can still be functional after incubation at 53^o^C for RT.

For proof-of-concept, we performed a pilot experiment (Figure S1B). We used a human iPSC line engineered with dox-inducible CRISPR activation (CRISPRa)-VPR and delivered 4 dual gRNA constructs into the cells by lentiviral transduction in an arrayed format. Each dual gRNA construct expresses 2 different activating guide sequences for the same target, in this case, 3 transcription factors (NEUROG2, GATA5, and MEOX1) and a non-targeting control (NT); guide sequences were selected from the top two performing guides in the Weissman V2 CRISPRa library as listed in Table S1. We activated transcription factors (TFs) in the pilot experiment to induce directed differentiation through overexpression of lineage-specific TFs,^25^ based in part on prior work that established sets of key regulators leading to directed differentiation in iPSCs.^26^^,^^27^ NEUROG2 is a well-established driver of neuronal cell fate,^28^ whereas GATA5 and MEOX1 overexpression caused loss of pluripotency markers in iPSCs^26^ but led to an undefined cell fate. By turning on the expression of these TFs in iPSCs through CRISPR-mediated activation, we expect the cells to exhibit changes in RNA and ATAC profiles that are distinct from those observed in the parental iPSCs, making this an ideal system to evaluate our CAT-ATAC method. After 7 days of dox induction, the cells were pooled and analyzed by CAT-ATAC. It is worth noting that excess cells were cryopreserved and used in subsequent iterations for optimization and no loss in performance was observed, demonstrating the flexibility and utility of this assay.

We obtained a total of 6,656 cells with both RNA and ATAC profiles after data preprocessing and filtering, of which 23% of cells could not be assigned to a gRNA identity, 30% of cells were assigned to a single gRNA, 35% of cells were assigned to a pair of protospacers (PS) with the same target, and 11% were classified as doublets that contained more than one gRNA with incongruous targets that likely resulted from droplets containing multiple cells (Figure 1B). The heatmap in Figure 1C depicts guide expression in all 6,656 cells with their gRNA identity labeled in each row. Upon filtering for only cells expressing paired gRNAs (*n* = 2,361 cells), we plotted the guide classification in a UMAP embedding (Figure 1D) and observed 4 distinct clusters corresponding to the 4 groups of cells receiving a different guide construct, indicating faithful gRNA identification. Figure 1E shows the RNA expression of the target genes on the guide UMAP with successful overexpression due to CRISPR-mediated gene activation. POU5F1 (gene name for Oct4) is a canonical iPSC marker, and its expression can be seen in the negative control gRNA cluster as well as the MEOX1 cluster, but it is reduced in NEUROG2 and GATA5. Violin plots in Figure S1C further demonstrate that TF upregulation is only observed in the cluster receiving the corresponding gRNAs, and POU5F1 is downregulated in both the GATA5 and NEUROG2 group, but not the MEOX1 group, suggesting that unlike cells in the former two groups, MEOX1 cells were not fully differentiated and may not yet have exited their pluripotent state, or may be on a lineage trajectory that requires sustained expression of POU5F1. Guide count rank plot in Figure 1F shows a median of 108 guide UMIs per cell. This is lower than the reported guide UMI of ∼1,000 in Perturb-seq^24^ due to differences in the assay chemistries and cell lines used but is sufficient to assign guide identity to a majority of the cells with high confidence, supported by the target gene expression data for each gRNA cluster.

Next, we proceeded with further evaluation of the pilot CAT-ATAC data by integrating both the RNA and ATAC profiles for each cell. Shown in Figure S1D, we performed clustering and constructed UMAP embeddings with just the RNA, ATAC, or weighted nearest neighbor (WNN) analysis.^29^ We classified the cells into 5 clusters, including one for each of the overexpressed TF, one for iPSCs, and another termed as intermediate due to the lack of expression of iPSC markers or targeted TFs. Motif enrichment was carried out for each cluster using Signac,^30^^,^^31^ and we observed a high level of correlation between RNA expression of the TF (in purple) and enrichment of its motif (in brown) (Figure 1G). Similarly, transcription factor footprinting analysis indicates increased Tn5 accessibility flanking the motif locations (Figure 1H). We compared CAT-ATAC to other recently published scRNA-seq, ATAC-seq and joint multiome technologies and found better performance of RNA and ATAC capture with our strategy, including increased RNA UMIs and genes per cell (Figures S1E and S1F) and increased ATAC fragments and peaks per cell (Figures S1G and S1H). This indicated that CAT-ATAC can successfully be used to analyze perturbation-specific effects on RNA and ATAC profiles of cells.

### Genome-wide CRISPRi screen identifies gene knockdowns that affect sensitivity to dasatinib

Next, we sought to integrate and apply the RNA and ATAC data to identify how genes affected by specific perturbations drive the mechanisms of cancer drug resistance and identify targets that can disrupt the associated transcriptional programs. For this application, we targeted CML resistance mechanisms to TKIs that are driven by activation of pathways independent of BCR-ABL mutations. We performed a genome-wide CRISPRi screen to identify genetic modifiers of sensitivity to the TKI dasatinib and hypothesized that some of these genetic modifiers must control transcriptional programs driving resistance.

**Figure 2:**
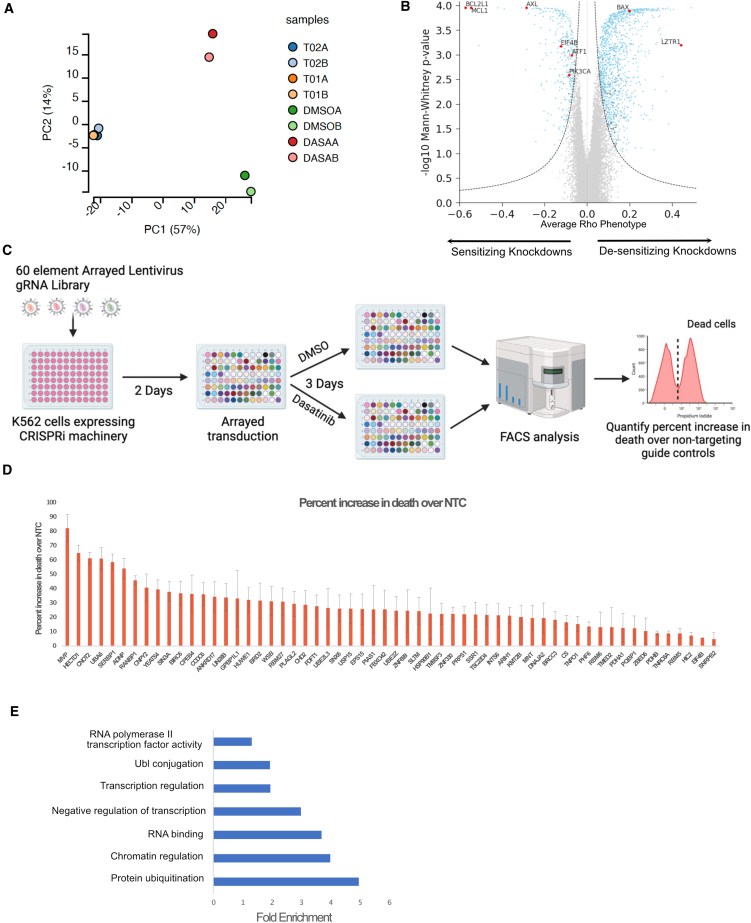
Pooled CRISPRi screen identifies gene knockdowns that modulate resistance to dasatinib (A) Principal-component analysis (PCA) plot showing reproducibility of replicate samples from the genome-wide screen. (B) Volcano plot showing gene knockdowns exhibiting sensitizing and desensitizing phenotypes. Blue dots represent all significant hits. Genes highlighted in red were previously known to affect dasatinib sensitivity. (C) Schematic showing overview of the arrayed validation experiment conducted to validate hits from the primary genome-wide screen. (D) Bar plot displaying results from the arrayed validation experiment showing percent increase in cell death for each of the guide expressing cells over non-targeting control guide expressing cells. Error bars represent standard deviation. (E) GO analysis for the 60 genes included in the arrayed validation experiment.

The screen was performed in K562 cells, a CML cell line that harbors a BCR-ABL mutation, and the experimental design is outlined in Figure S2A. After observing high concordance between the replicates (Figure 2A), we identified 164 dasatinib sensitizing gene knockdowns and 800 dasatinib de-sensitizing gene knockdowns that were differentially enriched using an adjusted *p* value cutoff of 0.05 and a phenotype score cutoff of ±0.1 (Figure 2B). These include previously reported TKI sensitizing genes BCL2L1, MCL1, and PI3K-AKT pathway genes as well as desensitizing genes BAX and LZTR1 (Figures 2B and S2B).^21^^,^^32^^,^^33^ We decided to focus on sensitizing gene hits as we hypothesized that some of these genes might constitute the cellular response to dasatinib and be responsible for resistance due to aberrant pathway activation. We filtered down the list of 164 sensitizing gene hits to 60 hits by first removing 61 genes that had significant growth phenotype scores in our vehicle control sample or which were identified as a core essential gene by Hart et al.^34^ and by keeping only the genes that have nuclear function to focus on potential core components of gene regulatory networks (Figure S2C). We performed arrayed validation for these 60 gene hits by transducing K562 cells expressing CRISPRi machinery with dual-guide constructs for the 60 genes (composed of the top two performing guides from the pooled survival screen) and 2 non-targeting controls (Figure 2C). Following fluorescence-activated cell sorting (FACS) analysis, we calculated percent increase in cell death and confirmed that all the gene knockdowns sensitized cells to dasatinib when compared to NTCs (Figure 2D). GO analysis indicated that these 60 genes were functionally classified as being involved in transcriptional regulation, protein ubiquitination and RNA binding (Figure 2E). Of the 20 genes with transcriptional control functions, there were 7 genes with known transcriptional repression function, 3 genes with known transcriptional activator function, and 10 genes with unknown transcriptional activity. The transcriptional networks controlled by these 20 genes could regulate cellular resistance to dasatinib and reveal new potential therapeutic targets. We hypothesized the global changes to the transcriptomic and DNA accessibility profiles after knockdown would shed light on the underlying regulatory networks and effectors that confer dasatinib resistance. Hence, we used CAT-ATAC to evaluate the effect of gene perturbations simultaneously on transcriptome and DNA accessibility from the same cells.

### Identification of perturbation-specific effects on dasatinib-induced alterations to DNA accessibility and transcription

**Figure 3:**
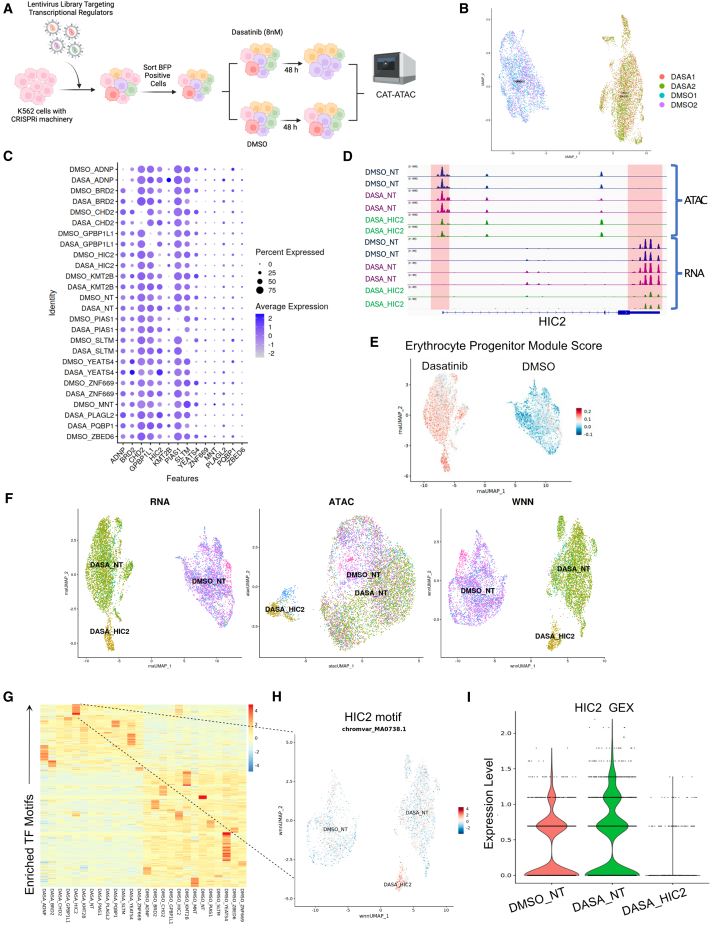
CAT-ATAC elucidates the combinatorial effect of gene and drug perturbation on RNA and ATAC profiles (A) Schematic showing the experimental outline for the CAT-ATAC experiment. (B) UMAPs after sample integration showing replicate reproducibility. (C) Dot plot showing target gene knockdown in cell populations that received the indicated guide in both dasatinib- and DMSO-treated conditions. (D) Normalized bigwig tracks for ATAC-seq and RNA-seq data for DMSO_NT, DASA_NT, and DASA_HIC2 cell groups at the HIC2 gene locus. Highlighted areas mark the promoter and 3′ end of the HIC2 gene. Scales on the *y* axis for ATAC-seq data are 0–3,000 units and for the RNA-seq data are 0–300 units. (E) Module score plot showing that dasatinib treatment results in upregulation of erythrocyte progenitors genes identified by Velten et al. (F) UMAPs showing RNA, ATAC, and weighted nearest neighbor profiles for cells expressing specific guide RNAs. (G) Heatmap showing drug treatment and knockdown-specific changes in chromvar motif activities. (H) UMAP showing motif enrichment for HIC2 in the cell groups DMSO_NT, DASA_NT, and DASA_HIC2. (I) Violin plots showing RNA expression of HIC2 in the cell groups DMSO_NT, DASA_NT, and DASA_HIC2.

K562 cells expressing CRISPR interference (CRISPRi) machinery were transduced with a pooled lentiviral library containing 26 pairs of gRNAs. The 26-element library comprised 20 dual-guide vectors targeting the 20 transcriptional regulatory genes identified previously and 6 non-targeting gRNA pairs. The gRNA sequences used are listed in Table S1. We harvested the cells 48 h after dasatinib treatment to minimize loss due to cell death and investigate the earliest transcriptional and DNA accessibility changes that happen upstream of cell death (Figure 3A). The experimental design consisted of 2 biological replicates resulting in 4 pooled CAT-ATAC samples, two treated with dasatinib and two treated with DMSO as a vehicle control. We processed the 4 samples individually using the Seurat^30^ analysis package and achieved a perturbation assignment rate of 72%–77% across the 4 samples (Figure S3A), and high concordance between the replicates in the UMAP (Figure 3B). After guide assignment and filtering, we observed significant knockdowns (median KD 85.12% ±10% SD) for all the targeted genes except for those where the percentage of cells expressing the target gene was very low (Figure 3C). Concomitantly, we saw a decrease in promoter accessibility for many of the targeted genes based on the scATAC data (Figures 3D and S3B).

Erythroid differentiation is one of the hallmarks of TKI treatment.^35^ To assess whether dasatinib treatment in the experiment produced the expected effect on cells, we used the genes identified by Velten et al. 2017^36^ to assess erythroid differentiation in response to dasatinib treatment. We calculated a module score for these genes and saw an enrichment of the module in the dasatinib-treated cells (Figure 3E) confirming the erythroid differentiation response and our ability to successfully assess transcriptional responses to dasatinib treatment in the context of genetic perturbations. When comparing populations with individual perturbations, we found that cells harboring knockdowns for the genes HIC2, KMT2B, YEATS4, PIAS1, PQBP1, and GPBP1L1 formed clusters when compared to the non-targeting guide expressing cells (Figure S3C). Among these genes, HIC2 knockdown had the strongest effect on both the transcriptome and ATAC-seq profiles especially in the dasatinib-treated group (Figure 3F), leading us to focus on identifying genes downstream of HIC2 that might confer resistance to dasatinib. ChromVar^37^ can take sparse chromatin accessibility data from single cells as input and predict enrichment of transcription factor motifs. Using ChromVar,^37^ we identified perturbation-specific enrichment of transcription factor motifs (Figure 3G). We assessed whether HIC2 demonstrates repressor activity,^38^ which has been previously reported. Transcriptional repressors exhibit anticorrelation between motif activity and gene expression,^39^ because chromatin bound by repressors is generally not accessible, and loss of repressor expression would expose more motifs normally occupied by the repressor. In agreement with these data, we find the HIC2 motif to be enriched in cells where HIC2 expression is downregulated with CRISPRi (Figures 3H and 3I). Having the paired RNA-seq and ATAC-seq data from the same single cells, along with perturbation identities, was essential in establishing the anti-correlative relationship between HIC2 expression and motif enrichment. We thus demonstrate that, when applied in specific cellular contexts, CAT-ATAC can reveal novel context-dependent activation and repressive behaviors of TFs.

### HIC2 indirectly activates genes that may cause dasatinib resistance

**Figure 4:**
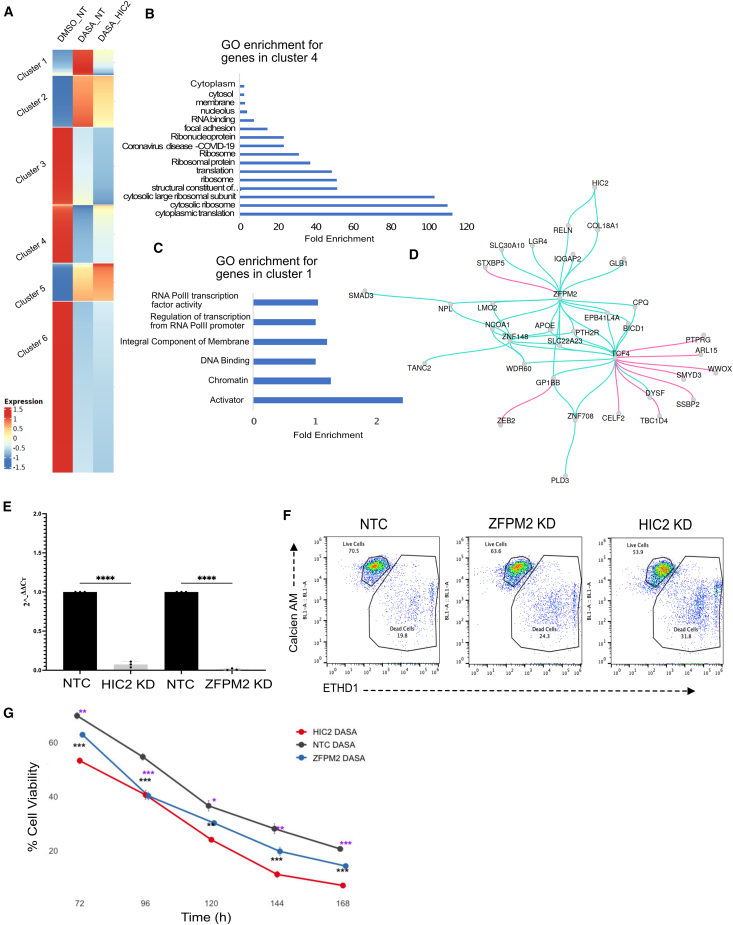
Identifying the gene regulatory network underlying dasatinib resistance (A) Heatmap showing gene expression patterns in DMSO-treated cells expressing non-targeting guides (DMSO_NT), dasatinib-treated cells expressing non-targeting guides (DASA_NT), and dasatinib-treated cells expressing HIC2-targeting guides (DASA_HIC2). (B) GO analysis for genes in cluster 4 from (A). (C) GO analysis for genes cluster 1 from (A). (D) Subsetted gene regulatory network identified by querying the global network for genes differentially downregulated in dasatinib-treated cells expressing HIC2 guides as compared to dasatinib-treated cells expressing NT guides. Cyan lines indicate activating interactions and the magenta lines indicate repressive interactions. (E) RT-qPCR data showing CRISPRi-mediated knockdown of gene expression for the genes HIC2 and ZFPM2. (F) Flow cytometry plots at 72 h post dasatinib treatment showing the distribution of cells for each of the three gene knockdown conditions along with control cells treated with dasatinib and stained with calcien-AM and EthD-1 to identify live and dead cells, respectively. (G) Time course experiment showing flow cytometry data from 72 to 168 h post-dasatinib treatment quantifying % viable cells in HIC2 KD, ZFPM2 KD, and control cells. Each data point represents the mean ± SD of three biological replicates (*n* = 3). Statistical significance was assessed at each time point using unpaired two-tailed t tests comparing HIC2 KD or ZFPM2 KD vs. NTC. Black asterisks indicate significant differences for HIC2 KD vs. NTC, whereas purple asterisks indicate significant differences for ZFPM2 KD vs. NTC (∗ corresponds to *p* < 0.05, ∗∗ corresponds to *p* < 0.01, and ∗∗∗ corresponds to *p* < 0.001).

Since both our genome-wide CRISPRi screen and CAT-ATAC data identified HIC2 as a significant hit, we went on to further identify genes controlled by HIC2 that drive dasatinib resistance. HIC2 knockdown cells formed a distinct cluster in the gene expression, ATAC, and joint UMAPs, suggesting there are broadly altered gene expression patterns under dasatanib treatment compared to non-targeting controls (Figure 3F), and leading us to hypothesize that the expression of HIC2 targets is altered upon treatment with dasatinib. Considering that HIC2 downregulation sensitizes cells to dasatinib, we hypothesized that some of the genes differentially expressed upon HIC2 knockdown should also affect sensitivity to dasatinib. Since HIC2 has repressor activity, the genes upregulated in the HIC2 knockdown cluster (DASA-HIC2 cells) would potentially be direct targets of HIC2. De-repression of these genes in K562 cells should sensitize them to dasatinib. For genes downregulated in the DASA-HIC2 condition, we expected that downregulation of these genes in K562s would sensitize the cells to dasatinib, and that their upregulation upon treatment with dasatinib could constitute the cellular resistance response. We plotted the mean normalized read counts for the differentially expressed genes in the three cell groups, DMSO-treated cells expressing non-targeting control gRNAs (DMSO_NT), dasatinib-treated cells expressing non-targeting control gRNAs (DASA_NT), and dasatinib-treated cells expressing HIC2 gRNAs (DASA_HIC2), and clustered them using k-means clustering to identify patterns of gene expression changes (Figures 4A; Table S2). We performed GO analysis for two sets of genes that follow up-down-up (cluster 4) and down-up-down (cluster 1) patterns of gene expression in the three conditions DMSO_NT, DASA_NT, and DASA_HIC2, respectively (Figures 4B and 4C). We observed that genes following the up-down-up pattern, which potentially include direct targets of HIC2, are mostly involved with processes exhibiting post-transcriptional control of gene expression (Figure 4B) while genes following a down-up-down pattern of gene expression, which may include dasatinib-resistance genes, were associated with transcriptional regulation functions (Figure 4C). CAT-ATAC can only infer transcriptional control mechanisms so we could not identify the genes controlled by direct targets of HIC2. Hence, we focused on identifying how the genes downregulated in the DASA-HIC2 cluster might contribute to dasatinib resistance via construction of gene regulatory networks.

### GRNs identify pathways involved in dasatinib resistance

Recent single-cell GRN inference methods use paired scRNA-seq and scATAC-seq data to infer direct regulatory relationships between genes.^40^^,^^41^^,^^42^ We used Pando^42^ to construct a gene regulatory network using scRNA and scATAC-seq data (Figure S4). We queried this network for the genes downregulated in the DASA-HIC2 cluster and identified a sub-network comprising first degree connections to the genes in the query list (Figure 4D). The cyan-colored connections show activating interactions while the magenta connections represent repressive interactions. Upon inspecting the expression levels of genes in the subnetwork, we found that majority of genes with activating interactions were upregulated in the DASA_NT cells as compared to DMSO_NT and DASA_HIC2 cells (Figure S5A), while genes with repressive interactions did not follow this pattern of expression (Figure S5B). We hypothesized that the genes with activating interactions in this sub-network are necessary for dasatinib resistance.

One such gene is ZFPM2, a transcription factor that is one of the central nodes in the sub-network in Figure 4D. The majority of interactions exhibited by ZFPM2 in this sub-network are activating interactions, leading us to hypothesize that ZFPM2 may be responsible for dasatinib resistance. To determine whether ZFPM2 is necessary for dasatinib resistance, we knocked down HIC2 (positive control) and ZFPM2 using CRISPRi in K562 cells (guide sequences in Table S1). Quantitative reverse-transcription PCR (RT-qPCR) analysis showed significant knockdown of gene expression for both genes when compared to non-targeting controls (Figure 4E). We next performed flow cytometry analysis post-dasatinib treatment to elucidate the effect of knockdowns on dasatinib sensitivity. The gating strategy for the flow cytometry analysis is illustrated in Figure S6A. After treating the cells with 8 nM dasatinib for 72 h, we observed that the cells with gene knockdowns showed a significant decrease in survival compared to the non-targeting control expressing cells, thereby indicating these genes caused increased resistance to dasatinib (Figure 4F). We also performed a longitudinal time course experiment quantifying percent viability of cells over time and saw significant decrease in viability for DASA-HIC2 and DASA-ZFPM2 cells across all the time points tested (Figure 4G). Thus, we can conclude that ZFPM2 confers dasatinib resistance.

### ZFPM2 may activate a putative enhancer upstream of TCF4

**Figure 5:**
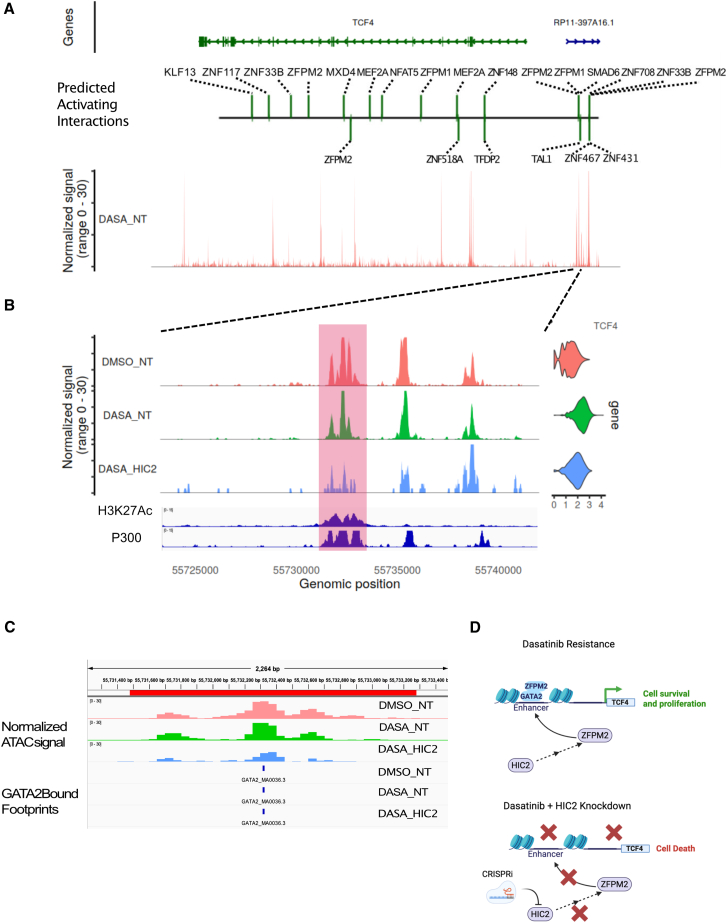
Analysis of chromatin accessibility data links ZFPM2 to TCF4 expression (A) Genome browser tracks showing the TCF4 locus. The first track shows the position of the TCF4 gene, the second track shows the activating interactions predicted at this locus by Pando, and the third track shows the ATAC-seq signal for the DASA_NT cell group at this locus. (B) Zoomed-in view of the region marked by dotted lines from (A). The highlighted region in this track shows an ATAC-seq peak exhibiting reduced accessibility in DASA_HIC2 cell group. The next two tracks showing H3K27Ac and P300 enrichment in K562 cells were downloaded from ENCODE. Violin plots show the gene expression of TCF4 in the three cell groups DMSO_NT, DASA_NT, and DASA_HIC2. (C) Genome browser tracks showing only the highlighted enhancer region from (B). First three tracks show the pseudobulked ATAC signal for the three cell groups DMSO_NT, DASA_NT, and DASA_HIC2. The last three tracks show the GATA2 bound footprints predicted using TOBIAS in the same three cell groups. (D) Proposed model for dasatinib resistance.

Our data indicate that one of ZFPM2’s activating interactors is the transcription factor TCF4. Upon inspecting the TCF4 locus, we identified an ATAC peak upstream of TCF4 (Figure 5A), which was less pronounced in the DASA-HIC2 cells (Figure 5B) and harbored a ZFPM2 activating interaction as predicted by Pando (Figures 5A and 5B). Decrease in accessibility of this peak correlated with a decrease in expression of the TCF4 gene in the DASA-HIC2 cell group (Figure 5B). We found that this peak overlaps with active histone marks H3K27Ac and P300 in K562 cells and is a potential active enhancer for TCF4 in K562 cells (Figure 5B). Publicly available HiC data for K562 cells from the 4D nucleosome consortium^43^ suggest that this upstream element and the TCF4 promoter lie within a contact domain with significant DNA-DNA contact frequency (Figure S6B) further suggesting that the upstream element could be a TCF4 enhancer.

We then wanted to identify how ZFPM2 might interact with this putative enhancer. It is unclear whether ZFPM2 binds DNA directly as there is no published ZFPM2 chromatin immunoprecipitation sequencing (ChIP-seq) data and no known ZFPM2 DNA binding motif. However, ZFPM2 is known to act as a co-factor for GATA TFs and can act as a context dependent transcriptional co-activator for GATA factors.^44^^,^^45^^,^^46^ GATA factors (especially GATA1, GATA2, and GATA3) are known to play a significant role in hematopoiesis,^47^^,^^48^ so we hypothesized that ZFPM2 might be interacting with the putative enhancer element upstream of TCF4 via these factors. We used FIMO^49^ to test the occurrence of GATA binding motifs in the putative enhancer element using position weight matrices from three databases JASPAR, CIS-BP, and HOCOMOCO and found significant occurrence of GATA1, GATA2, and GATA3 motifs in this element (Table S3). Using pseudobulked ATAC data from the three cell groups DMSO_NT, DASA_NT, and DASA_HIC2, we performed footprinting analysis with the TOBIAS^50^ suite of tools and found protected GATA2 footprints at the putative enhancer element (Figure 5C). We thus hypothesize that ZFPM2 might bind the putative TCF4 enhancer via GATA2 and thereby control TCF4 expression. TCF4 is an effector for the WNT signaling pathway,^51^ which is known to be activated in the context of TKI resistance.^52^^,^^53^^,^^54^ Thus, ZFPM2 may activate WNT downstream targets by controlling TCF4 expression, thereby conferring dasatinib resistance. Gene set enrichment analysis further confirmed enrichment of WNT pathway genes in DASA_NT cells when compared with DASA_HIC2 cells (Figures S7A and S7B). We propose a model whereby dasatinib resistant cells show a HIC2 dependent ZFPM2 upregulation that partners with GATA2 to activate a putative TCF4 enhancer, further activating WNT pathway genes and assisting cell survival (Figure 5D). Information about perturbation status of the target gene, ATAC-seq and RNA-seq data from the same cell allowed us to determine that HIC2 is necessary for ZFPM2 expression, which in turn might contribute to activating TCF4 gene expression as a WNT pathway effector necessary for cell survival when treated with dasatinib. This paired data derived from CAT-ATAC allowed for the identification of a *cis*-regulatory relationship between the TCF4 promoter and an accessible DNA site upstream of TCF4. CAT-ATAC thus provides the ability to build testable mechanistic hypotheses by combining multiple data types from different aspects of cellular regulatory processes.

## Discussion

We demonstrate that CAT-ATAC robustly captures gRNAs, achieving up to 77% perturbation assignment rate in tested cell lines, while producing high-quality scRNA- and scATAC-seq data. This method lays the foundation for a powerful new platform for CRISPR screening that integrates small-scale to genome-wide perturbations with single-cell resolution of both gene expression and chromatin accessibility. By jointly profiling these modalities in the same cells, CAT-ATAC eliminates the need for separate pooled screens and minimizes technical and biological confounders.

Our implementation uses a primer containing the CS1 direct capture sequence to reverse transcribe gRNAs (Figure 1A), but CAT-ATAC is inherently flexible. It can accommodate a wide range of CRISPR perturbations or synthetic constructs by incorporating a splinted reverse transcription primer targeting the desired sequence. While recent studies have introduced alternative approaches to perform CRISPR screens with multimodal gene expression and open chromatin readouts,^55^^,^^56^ they typically require either custom vector engineering^55^ or implementing complicated experimental approaches with multiple rounds of barcoding.^56^ In contrast, CAT-ATAC offers a straightforward, accessible workflow with only minimal modifications to the widely used 10× Genomics Multiome assay. It is compatible with standard CRISPR gRNA libraries and delivers strong capture efficiency, which can likely be enhanced further by employing enrichment strategies such as biotinylated PCR primers, as demonstrated in MultiPerturb-seq.^56^ Additionally, the ability to directly capture gRNAs enables the use of constructs that express multiple gRNAs targeting one or several genes,^23^^,^^57^^,^^58^ which makes the studies of combinatorial perturbations feasible.

Using this platform, we performed a chemical-genetic screen and identified genetic modifiers that sensitize cells to dasatinib, including regulators of transcription. Notably, we discovered that HIC2 expression confers resistance to dasatinib—representing the first direct functional evidence linking HIC2 to drug response. This finding is consistent with prior reports of HIC2 overexpression in CD34^+^ cells from CML patients, suggesting a broader role in disease progression.^59^

By leveraging the multimodal data generated with CAT-ATAC, we uncovered a gene regulatory network activated under dasatinib treatment that depends on HIC2 expression and identified ZFPM2 as a central node in this network. The paired scATAC- and scRNA-seq data from HIC2 knockdown cells enabled prediction of a mechanism whereby ZFPM2 and GATA2 bind a *cis*-regulatory element upstream of TCF4 to control expression of TCF4, a transcription factor known to be an effector for the WNT signaling pathway that contributes to TKI resistance in CML and other myeloid leukemias. Although the enhancer-gene relationship was inferred through motif analysis and chromatin accessibility, and further validated by publicly available Hi-C data, future experimental work—such as ChIP-seq or enhancer perturbation—could provide confirmation of this regulatory link. This example illustrates the power of CAT-ATAC to identify novel candidate regulatory interactions at single-cell resolution and highlights the value of integrated chromatin and transcriptomic profiling for dissecting complex gene networks.

Beyond CRISPR screens, the versatility of CAT-ATAC makes it broadly applicable to capturing any nucleic acid of interest—from synthetic reporters and immune receptor sequences to antibody-tagged oligonucleotides. These types of multiparametric single-cell readouts will be transformative to shape our understanding of fundamental biological processes, cell fate decisions, and disease mechanisms by harnessing the heterogeneity inherent to complex cell populations instead of reducing datasets to bulk analysis. By providing a better understanding of the link between DNA accessibility, gene transcription, and protein translation and processing, these approaches can be broadly applied to probe regulation at non-coding regions of the genome, allowing for more systematic studies of the links between gene regulatory elements and gene expression. In addition, these tools can be leveraged to gain biological and mechanistic insights into the relationship between recognition motifs, transcription factor binding, and downstream gene activity, enabling further exploration of the dynamics of transcription factor-driven cell fate changes. Furthermore, these types of multimodal perturbation datasets will prove invaluable for building foundational models in biology such as the virtual cell.^60^

### Limitations of the study

As with all single-nucleus based methods, the 10× Genomics Multiome assay introduces certain limitations that are important to consider. Nuclear RNA represents only a subset of the total cellular transcriptome, often biased toward unspliced and nascent transcripts, which may affect sensitivity for some gene expression readouts. Additionally, detection of CRISPR gRNAs from nuclear preparations can be variable, depending on factors such as guide stability and nuclear localization. Nuclear preparations also increase the amount of ambient RNA in the reaction, as the cells release cytosolic RNA upon lysis that affects the guide to cell assignment rate. Importantly, nuclei isolation is intrinsic to the multiome platform, as it enables concurrent ATAC-seq profiling, and our approach is designed within these technical constraints. Despite these limitations, the method provides a scalable and versatile platform for joint transcriptomic and epigenomic analysis in perturbation contexts, enabling high-resolution inference of regulatory mechanisms.

## Resource availability

### Lead contact

Requests for further information and resources should be directed to and will be fulfilled by the lead contact, Eric D. Chow (eric.chow@ucsf.edu).

### Materials availability

Plasmid vector backbone pLGR134 generated in this study are available from the lead contact with a completed materials transfer agreement.

### Data and code availability

• Raw and processed CAT-ATAC sequencing data can be downloaded from Gene Expression Omnibus (GEO: GSE288996). Data from previously published studies were from the Sequence Read Archive or Gene Expression Omnibus: SHARE-seq^61^ (GEO: GSE140203), SNARE-seq^62^ (GEO: GSE126074), Paired-seq^63^ (GEO: GSE130399), sci-CAR-seq^64^ (GEO: GSE117089), and MultiPerturb-seq^56^ (GEO: GSE277747). The human genome hg38 (GENCODE v32/Ensembl98) was from 10× Genomics (https://cf.10xgenomics.com/supp/cell-exp/refdata-gex-GRCh38-2020-A.tar.gz).
• All the code generated for analysis of the CAT-ATAC data including for counting guides, guide to cell assignment and gene regulatory analysis is available on GitHub (https://github.com/ucsf-lgr/catatac_public) and on Zenodo using the DOI https://doi.org/10.5281/zenodo.17238053
• Any additional information required to re-analyze the data reported in this paper is available from the lead contact upon request.

## Acknowledgments

We thank the UCSF Center for Advanced Technology (CAT) for NGS sequencing services. Sequencing was performed at the UCSF CAT, supported by UCSF PBBR, RRP IMIA, and NIH 1S10OD028511-01 grants. We would also like to thank LGR team members Greyson Lewis, Sailaja Peddada, and Adam Litterman for their advice and Lauren Enriquez for help in setting up automation protocols for guide cloning. Some figure elements were created using BioRender.com. This work is supported by the Laboratory for Genomics Research established by GSK, UCSF, and UC Berkeley.

## Author contributions

Conceptualization, K.S., Y.A.Y., E.D.C., and L.P.; data curation, K.S., Y.A.Y., K.F., S.F., and V.S.; formal analysis, K.F., K.S., Y.A.Y., V.S., J.P., and S.F.; funding acquisition, K.S., L.P., and E.D.C.; investigation, K.S., Y.A.Y., K.M., G.A., R.M., and R.E.; methodology, Y.A.Y., E.D.C., K.S., and R.E.; project administration, L.P., E.D.C., and S.S.; resources, L.P. and S.S.; software, K.S., Y.A.Y., K.F., V.S., and S.F.; supervision, K.S., Y.A.Y., L.P., and E.D.C.; validation, K.S., H.E., and K.M.; visualization, K.F., K.S., E.D.C., L.P., and Y.A.Y.; writing – original draft, K.S.; writing – review & editing, K.S., L.P., E.D.C., and Y.A.Y.

## Declaration of interests

Y.A.Y., V.S., J.P., R.E., G.A., and S.S. are employees of GSK. E.D.C. is a co-founder of Survey Genomics.

## STAR★Methods

### Key resources table

**Table:** 

REAGENT or RESOURCE	SOURCE	IDENTIFIER
**Bacterial and virus strains**		

DH5-Alpha high efficiency competent E. Coli	NEB	Cat# C2987U

**Chemicals, peptides, and recombinant proteins**		

BsmBI	NEB	Cat# R0739S
mTeSR1	StemCell Technologies	Cat# 85850
FBS	VWR	Cat# 97069–085
Accutase	ThermoFisher Scientific	Cat# A1110501
Polybrene	Sigma	Cat# TR-1003-50ul
Puromycin	ThermoFisher Scientific	Cat# A1113803
Doxycycline	Sigma	Cat# D5207
TransIT®-293 Transfection Reagent	Mirus Bio	Cat# MIR 2700
TransIT-VirusGEN® Reagent	Mirus Bio	Cat# MIR 6704
Dasatinib	Millipore Sigma	Cat# SML2589
DMSO	Sigma	Cat# D8418
Calcien-AM	ThermoFisher Scientific	Cat# C1430
Ethidium Homodimer-1	ThermoFisher Scientific	Cat# E1169
Hi-T4 ligase	NEB	Cat# M2622S
Kapa HiFi HotStart ReadyMix	Roche	Cat# KK2601

**Critical commercial assays**		

Macherey-Nagel Nucleospin Blood XL kit	Macherey-Nagel	Cat# 740950
Single Cell Multiome ATAC + Gene Expression	10× Genomics	Cat# 1000283
RNeasy Plus Mini Kit	Qiagen	Cat# 74134
SuperScript Vilo cDNA synthesis kit	ThermoFisher Scientific	Cat# 11754050
SYBR-green qPCR master mix	ThermoFisher Scientific	Cat# 43-676-59
Lenti-X™ Concentrator	Takara Bio	Cat# 631232

**Deposited data**		

CAT-ATAC data for both the iPSC and K562 experiments	This Paper	GSE288996
SHARE-seq		GSE140203
SNARE-seq		GSE126074
Paired-seq		GSE130339
sci-CAR-seq		GSE117089
MultiPerturb-seq		GSE277747

**Experimental models: Cell lines**		

Human iPSC	N/A	N/A
K562 line with CRISPRi machinery	Gift from the Gilbert lab	N/A
LentiX	Takara	Cat# 632180
HEK 293T	ATCC	Cat# CRL-3216

**Oligonucleotides**		

RT primer CS1_12bp UMI:/5Phos/TCGTCGGC AGCGTCAGATGTGTATAAGAGACAGNNNNN NNNNNNNTTGCTAGGACCGGCCTTAAAGC	This paper	N/A
splint with LNA: TGACGCTGCC+G+A + C + G + ACAGACGCG/3Phos/	This paper	N/A
R2-TSO primer: GTGACTGGAGTTCAGACGT GTGCTCTTCCGATCTAAGCAGTGGTATCAACGCAGAG	This paper	N/A
Partial P5 forward: AATGATACGGCGACCACCGAGA	This paper	N/A
P7-i7-R2 primer A1: caagcagaagacggcatacgagat TTCGCAGTgtgactggagttcagacgtgtgctcttccgatct	This paper	N/A
P7-i7-R2 primer A2: caagcagaagacggcatacgagat CGAGACTAgtgactggagttcagacgtgtgctcttccgatct	This paper	N/A
P7-i7-R2 primer A3: caagcagaagacggcatacgagat ACAGCTCAgtgactggagttcagacgtgtgctcttccgatct	This paper	N/A
P7-i7-R2 primer B1: caagcagaagacggcatacgagat AAGTGTCGgtgactggagttcagacgtgtgctcttccgatct	This paper	N/A
P7-i7-R2 primer A1_1: caagcagaagacggcatacgaga tAGTAAACCgtgactggagttcagacgtgtgctcttccgatct	This paper	N/A
P7-i7-R2 primer A1_2: caagcagaagacggcatacgagat CCGTTTAGgtgactggagttcagacgtgtgctcttccgatct	This paper	N/A
AGACTCACACGGAGGAAGAGCT	This paper	HIC2 human PCR forward primer
GTCTTCTCGCAGACCGAACACT	This paper	HIC2 human PCR reverse primer
GTGACTTGGCAAGGAGTGGAAG	This paper	ZFPM2 human PCR forward primer
CATCTGACTGGCAGCTTGTAGC	This paper	ZFPM2 human PCR reverse primer
CAGCCTCAAGATCATCAGCA	This paper	GAPDH human PCR forward primer
TGTGGTCATGAGTCCTTCCA	This paper	GAPDH human PCR reverse primer

**Recombinant DNA**		

pLGR002 dual-guide CRISPRi vector backbone	Addgene	188320

**Software and algorithms**		

BBDuk	https://archive.jgi.doe.gov/data-and-tools/software-tools/bbtools/	N/A
Cell Ranger ARC	https://www.10xgenomics.com/support/software/cell-ranger-arc/latest	N/A
Seurat	https://satijalab.org/seurat/	N/A
HTOdemux	N/A	N/A
Signac	https://stuartlab.org/signac/	N/A
Pando	https://github.com/quadbio/Pando	N/A
Subset-bam	https://github.com/10XGenomics/subset-bam	N/A
TOBIAS	https://github.com/loosolab/TOBIAS	N/A

### Experimental model and study participant details

Human cell lines were sourced ethically, and their research use was in accord with the terms of the informed consent under an IRB/EC approved protocol. iPSC cell line (Proprietary for GSK use generated by Takara) was engineered to express doxycycline inducible CRISPRa machinery (dCas9-VP64-p65-Rta). The CRISPRa machinery was integrated into the AAVS1 safe harbor locus to minimize silencing.

The K562-CRISPRi cell line was a gift from the Gilbert lab. K562-CRISPRi line was maintained in the standard conditions of DMEM+10% FBS with antibiotics as recommended by ATCC.

### Method details

#### gRNA cloning

For the pilot experiment top two guide RNAs from the Weissman V2 CRISPRi library targeting the 3 transcription factors (GATA1, MEOX1 and NGN2) and were cloned in dual guide lentiviral construct compatible with Perturb-seq using Gibson assembly. For the arrayed validation of hits from the dasatinib screen top two guides that exhibited the highest phenotype score for sensitizing the cells to dasatinib were cloned individually in the dual guide gRNA vector using Gibson assembly. Gibson assembly process was as follows:

Dual guide RNAs were cloned in a lentiviral vector backbone using Gibson assembly cloning. An insert DNA sequence with flanking BsmBI restriction sites and containing a constant region with Perturb-seq compatible capture sequence 1 (CS1: GCTTTAAGGCCGGTCCTAGCAA) was cloned into an intermediate transfer vector and transformed into bacteria for plasmid amplification (See insert sequence below). To prepare a linear insert for cloning the amplified plasmid was digested using BsmBI restriction digestion and the desired fragment was purified using gel purification. The backbone vector was also linearized using BsmBI restriction digestion. This backbone vector (pLGR134) was derived from pLGR002 (Addgene #188320) by swapping out the BstXI and BlpI sites for a pair for BsmBI sites. ssDNA oligos for guides were ordered with 20 base pair overhangs on both 5′ and 3′ sides that partially overlap the constant region and mU6 promoter for guide 1 and constant region and hU6 promoter for guide 2. Linearized vector, insert and guide oligos were then assembled into dual guide vectors in a Gibson assembly reaction. Colonies were validated by Sanger sequencing.

#### Insert sequence before BsmBI restriction digest

CGTCTCAagaggtttcAGAGCTAAGCACAAGAGTGCATAGCAAGTTGAAATAAGGCTAGTCCGTTTACAACTTGGCCGCTTTAAGGCCGGTCCTAGCAAGGCCAAGTGGCACCCGAGTCGGGTGCTTTTTTTGCTCGAATCTACACTCAGCTATGGCGCGCCCCAAGGTCGGGCAGGAAGAGGGCCTATTTCCCATGATTCCTTCATATTTGCATATACGATACAAGGCTGTTAGAGAGATAATTGGAATTAATTTGACTGTAAACACAAAGATATTAGTACAAAATACGTGACGTAGAAAGTAATAATTTCTTGGGTAGTTTGCAGTTTTAAAATTATGTTTTAAAATGGACTATCATATGCTTACCGTAACTTGAAAGTATTTCGATTTCTTGGCTTTATATATCTTGTGGAAAGCCAgaaacatgGAAAGGAGACG

Plasmids for the vectors targeting the 20 selected genes along with 6 NTCs required for the pooled multiome screen were then mixed in equimolar amounts to generate a pooled plasmid library.

#### Lentivirus production and transduction

For all arrayed experiments lentivirus was generated in an arrayed format by transfecting LentiX cells (Takara 632180) with individual gRNA vectors. To generate the lentivirus for transducing iPSC cells LentiX cells were forward transfected with the gRNA vectors and lentiviral packaging plasmids (dR8.91 and MD2G) using the Mirus transfection reagent (MIR 2700). The three plasmids were mixed in 1:1:0.1 ratios for gRNA vector, MD2G and dR8.91 respectively. The lentivirus was then harvested 48h post transfection in MTeSR1 medium for transduction in iPSCs. The lentivirus for arrayed validation experiment was generated in a 96 well format where the LentiX cells were reverse transfected using TransIT-VirusGEN Reagent (MIR 6704). The gRNA and packaging plasmids were mixed in the same 1:1:0.1 ratio followed by lentivirus harvest in media with 10% FBS. Pooled lentiviruses were generated in either 6 well or 15 cm dishes depending on the amount of lentivirus required. LentiX cells were forward transduced using Mirus transfection reagent and the plasmids were mixed in the ratios mentioned above. Lentivirus was then harvested in media containing 10% FBS.

#### Genome-wide CRISPRi screen

K562 cells containing dCas9-KRAB CRISPRi machinery were infected with CRISPRi V2 library containing the top 5 guides (Addgene #83969) to achieve 1000-fold coverage at an MOI of 0.3. Transduced cells were selected with puromycin until 90% of the cell population showed BFP expression indicating guide expression. Cells were then treated with a single dose of 8nM dasatinib (determined from a drug titration experiment) for 72h. This was followed by recovery for 5 days. An equivalent number of transduced cells were treated with DMSO as a vehicle control. Both the dasatinib and DMSO treated cells were then collected and genomic DNA was isolated using the Macherey-Nagel Nucleospin Blood XL kit (740950). The cassette encoding the gRNAs was then amplified by PCR and gRNA abundance was determined by next generation sequencing (NGS) as previously described.^65^^,^^66^^,^^67^ The libraries were sequenced on an Illumina NextSeq550 sequencer and the sequencing data were analyzed using the screen processing pipeline.^65^

#### Arrayed validation of the hits from CRISPRi screen

A dual-guide lentiviral library targeting 60 genes, and two non-targeting controls was prepared in-house as described above and used for arrayed lentiviral generation in HEK 293T cells. K562-CRISPRi cells were then “spinfected” at 1000 RCF for 1 h at 22C with this lentivirus in an arrayed fashion in the presence of 1 μg/mL of polybrene, left overnight at 37C, and then selected via puromycin at a concentration of 2ug/mL for 48 h. Following selection, guide-infected cell lines were treated with 8nM Dasatinib and their viability was recorded using Propidium Iodide nuclear stain (Invitrogen P1304MP) every 24 h following treatment on an Invitrogen Attune NxT Flow Cytometer.

#### CAT-ATAC

##### CAT-ATAC pilot experiment in iPSCs

iPSCs were transduced at MOI of 0.3 in the presence of 1 μg/mL of polybrene overnight in an arrayed format, followed by puromycin selection for one week at a concentration of 2 μg/mL. Three transcription factors were overexpressed (NEUROG2, GATA5, MEOX1) by supplementing doxycycline (4uM) in the media (mTeSR1, StemCell Technologies) for 7 days prior to CAT-ATAC analysis. As a control, a non-targeting scrambled gRNA sequence was used. After 7 days of overexpression cells were dissociated into a single-cell suspension using Accutase (ThermoFisher Scientific A1110501), counted and then equal numbers of cells per perturbation were mixed to make a pool of cells prior to processing for CAT-ATAC.

#### Pooled lentivirus generation and dasatinib treatment

A pooled lentiviral library containing 26 dual-guide vectors targeting 20 previously identified transcriptional regulatory genes and 6 non-targeting genes was generated as described previously. The resulting virus was then titered to determine the amount of virus required for desired MOI of 0.1 in K562 cells. Accordingly, 1.5∗10ˆ7 cells containing CRISPRi machinery were spun down and resuspended directly into 2.4mL of this pooled virus and “spinfected” at 1000 RCF for 1 h at 22C in the presence of 1 μg/mL of polybrene and incubating overnight at 37C. Guide infected cells were sorted via FACS based on BFP expression and allowed to recover for 5 days 2.5∗10ˆ7 of these sorted cells were then aliquoted into 4 shaking flasks, two of which were treated with Dasatinib and two of which were treated with DMSO for 48 h before being collected for CAT-ATAC.

#### CAT-ATAC method

Detailed CAT-ATAC protocol is in Supplemental Methods S1. Briefly, cells were harvested, nuclei were extracted and tagmented according to the 10x Multiome protocol. During the GEM Generation step, a duplex splint oligo is spiked into the barcoding reaction mix to allow reverse transcription of gRNAs and ligation onto the capture oligo of 10x Multiome gel beads. The resulting cDNA from captured guide RNAs is amplified to make an Illumina-compatible sequencing library containing P5 and P7 adaptors as well as sample indexes. GEX libraries were sequenced on a NovaSeqX with paired-end sequencing, 28 × 10 × 10 × 90, at a minimum of 20,000 read pairs/cell. ATAC and CRISPR libraries were pooled at a 5:1 ratio and were sequenced on NovaSeqX with paired-end sequencing, 151 × 8 × 24 × 151 (since at least 75 cycles of sequencing are required in read 2 to sequence through protospacer region for CRISPR libraries), at a minimum of 25,000 read pairs/cell for ATAC libraries and 5,000 read pairs/cell for CRISPR libraries.

#### CAT-ATAC data analysis

##### Processing FASTQ files after NGS

We extracted the protospacer-cell barcode pairs from the guide capture FASTQ files using a custom Bash script that first finds the reads containing both the protospacer and capture sequence via BBDuk alignment (https://sourceforge.net/projects/bbmap/) at a Hamming distance of 1. The matching reads are then demultiplexing based on unique UMIs using a guide calling algorithm described in the guide assignment section, and count tables are generated. GEX and ATAC FASTQs were aligned to GRCh38 version of the human genome using prebuilt packages for Cell Ranger ARC v.2.0.1. Post alignment filtering, barcode counting and counting of GEX and ATAC molecules in the FASTQ files was performed using the cellranger-arc count function.

#### Guide assignment

For the pilot CRISPRa experiment in iPSCs cells were assigned a guide identity using HTOdemux function in Seurat that is based on a previously published algorithm that was developed for cell hashing oligos.^68^ We found that this method had a low signal to noise ratio in our pooled CRISPRi screen, so we developed a new algorithm to assign guides to cells. For the pooled CRISPRi CAT-ATAC screen the UMIs per cell for each guide are modeled using a mixture of a zero-inflated negative binomial distribution and a negative binomial distribution (without zero-inflation). To maintain identifiability, the parameter space is constrained so that the negative binomial distribution without zero inflation has a larger mean than the negative binomial component of the zero-inflated distribution. Estimates for the negative binomial parameters and mixture proportions were obtained by maximum likelihood and used to determine the posterior probability that each observation arose from the negative binomial component with the larger mean. A cell was classified as expressing a guide if the posterior probability that the corresponding UMI measurement came from the negative binomial component with the larger mean was greater than 0.5.

We used the same strategy as reported by Replogle et al. 2022^23^ to further select cells for downstream analysis whereby cells bearing either one guide or two guides targeting the same gene were used for downstream analysis.

#### H3K27Ac and P300 ChIPseq data

Bigwig files for H3K27Ac (ENCSR000AKP) and P300 (ENCSR000EGE) ChIPseq datasets in K562 were downloaded from ENCODE (The Encyclopedia of DNA Elements) consortium.

#### Gene Ontology analysis

Selected gene sets either identified as hits in the CRISPR screens or as significantly differentially expressed genes were used for gene ontology analysis as mentioned in the results section. Gene ontology analysis was performed using the DAVID knowledgebase (https://davidbioinformatics.nih.gov/).

#### Single cell motif enrichment and footprinting analysis

Motif and footprinting analysis were performed using Signac’s AddMotifs, FindMotifs, and FootPrint functions. Motif activities were calculated using Signac’s RunChromVAR function and JASPAR2024 database.^69^

#### Pseudobulk ATAC analysis

Separate pseudobulked bam files were created for each of the cell groups DMSO_NT, DASA_NT and DASA_HIC2 using the 10x Genomics’ subset-bam tool. These individual bam files were then processed following the TOBIAS^50^ tools ATACorrect, ScoreBigwig and BINDetect to estimate the GATA2 bound footprints from the pseudobulked ATAC data.

#### Gene regulatory network inference with pando

To perform gene regulatory network inference, we used the functions initiate_grn, find_motifs, infer_grn, find_modules, and NetworkModules from the Pando package.^42^ We first built a global GRN including all the cells in the integrated multiome object. To identify the HIC2 specific network we subsetted this global GRN using a list of genes that were differentially downregulated in DASA_HIC2 cells as compared to DASA_NT cells.

#### Validation of ZFPM2

The top two guide RNAs from the Weissman V2 CRISPRi library targeting ZFPM2 gene were cloned into the LGR dual guide vector as described earlier. Lentivirus was generated as described before and concentrated using Lenti-X Concentrator (cat # 631232). K562 cells containing CRISPRi machinery were then transduced with guide lentiviruses for HIC2 (positive control), a non-targeting control (NTC) and ZFPM2. The transduced cells were selected with puromycin and then used for downstream validation experiments.

#### Validation of target knockdown post CRISPRi

Total RNA was extracted from K562 CRISPRi constructs targeted against NTC, HIC-2, and ZFPM2 (*n* = 3/each group) by using RNeasy Plus Mini Kit (Cat # 74134) and converted to cDNA using SuperScript Vilo cDNA synthesis kit (Cat # 11754050). A total of 1 μg RNA was converted to cDNA. cDNA was amplified using an SYBR-green qPCR master mix (Cat #43-676-59) and specific primers (key resources table). GAPDH was used as the reference gene.

The PCR steps consisted of an initial activation 50°C for 2 min, heat activation step at 95°C for 10 min, denaturation of at 95°C for 15 s, followed by annealing step at 60°C for 1 min for 40 cycles. The last step is the melting curve (95°C for 15 s, 60°C for 15 s, and 95°C for 15 s). Detection, quantification, and data analysis were performed in the CFX manager real-time detection system (Bio-Rad). The Ct values were calculated for each gene and were compared with the reference gene GAPDH, followed by estimation of ΔΔCt and fold change (2^−ΔΔCt^) to assess the relative gene expression.

#### Dasatinib treatment and cell viability analysis

Dasatinib (Millipore Sigma, cat. # SML2589) was diluted with DMSO to final concentration of 8nM, 0.01% DMSO was used as a vehicle control. K562 cells expressing either NTC, HIC2 or ZFPM2 guides were treated with 8nM dasatinib for 72 h. Live-dead staining was performed using 2uM calcien-AM (cat #C1430) and 2.5uM ETHD1 EthD-1 (cat #E1169) and cells were analyzed using the Attune NextGen flow cytometer (Thermo Scientific, USA) at 72h, 96h, 120h, 144h and 168h post dasatinib treatment. FSC and SSC dot plot was used to gate out the debris present in the suspension and percentages of live (calcien-AM positive) and dead (EthD-1 positive) cells were estimated.

### Quantification and statistical analysis

#### Data QC, filtering, integration and differential testing

Seurat v.4.3.0^30^ was used for single cell analysis. Cells with mitochondrial RNA percentage >20%, RNA count <1000 and >40000, nucleosome signal >2, and TSS enrichment <1 were filtered out. Seurat SCTransform v2.0 was used to normalize and scale the data as well as to find variable features. Cell cycle-associated genes were also regressed out using Seurat’s CellCycleScoring SCTransform functions to mitigate their effects on downstream analyses. We then ran Mixscape on each sample to calculate perturbation-specific signatures for each cell. This step included the identification and exclusion of cells that evaded CRISPR-mediated perturbation. The method also facilitated the visualization of similarities and differences among cells subjected to various perturbations.

The top 3,000 highly variable genes were used for PCA and UMAP dimensionality reduction. Libraries were integrated using Seurat’s merge and IntegrateEmbeddings functions. Cells were clustered using the FindClusters function and the Louvain algorithm. To identify significant changes associated with each perturbation, differential expression testing was conducted using Wilcoxon rank-sum test through Seurat FindMarkers function and differential accessibility testing was conducted using logistic regression in Signac.

#### Module score analysis

Genes associated with erythroid differentiation were identified from Velten et al.^36^ Module scores for these sets of genes were calculated using the AddModuleScore function in Seurat. These module scores were then plotted on the rnaUMAP to visualize the enrichment of the module in specific treatment groups.

## Supplemental information

Document S1. Figures S1–S7, and Method S1

Table S1. Sequences of guide RNAs used in the study

Table S2.Cluster identity for the genes represented in the heatmap in Figure 4A, related to Figure 4

Table S3. GATA motif occurences using FIMO

Document S2. Article plus supplemental information
