# Supplementary material for: Simultaneous capture of single cell RNA-seq, ATAC-seq, and CRISPR perturbation enables multiomic screens to identify gene regulatory relationships
Source: Cell Rep Methods. 2025 Nov 10;5(12):101222. doi: 10.1016/j.crmeth.2025.101222 (PMC12859477; doi:10.1016/j.crmeth.2025.101222)
Supplement: Document S1. Figures S1–S7, and Method S1 [file mmc1.pdf]

Cell Reports Methods, Volume *5*

Supplemental information

Simultaneous capture of single cell RNA-seq, ATAC-seq, and CRISPR perturbation enables multiomic screens to identify gene regulatory relationships

Kaivalya Shevade, Yeqing Angela Yang, Kevin Feng, Karl Mader, Volkan Sevim, Jacob Parsons, Gunisha Arora, Hasnaa Elfawy, Rachel Mace, Scot Federman, Rustam Esanov, Shawn Shafer, Eric D. Chow, and Laralynne Przybyla

Supplementary Figure 1

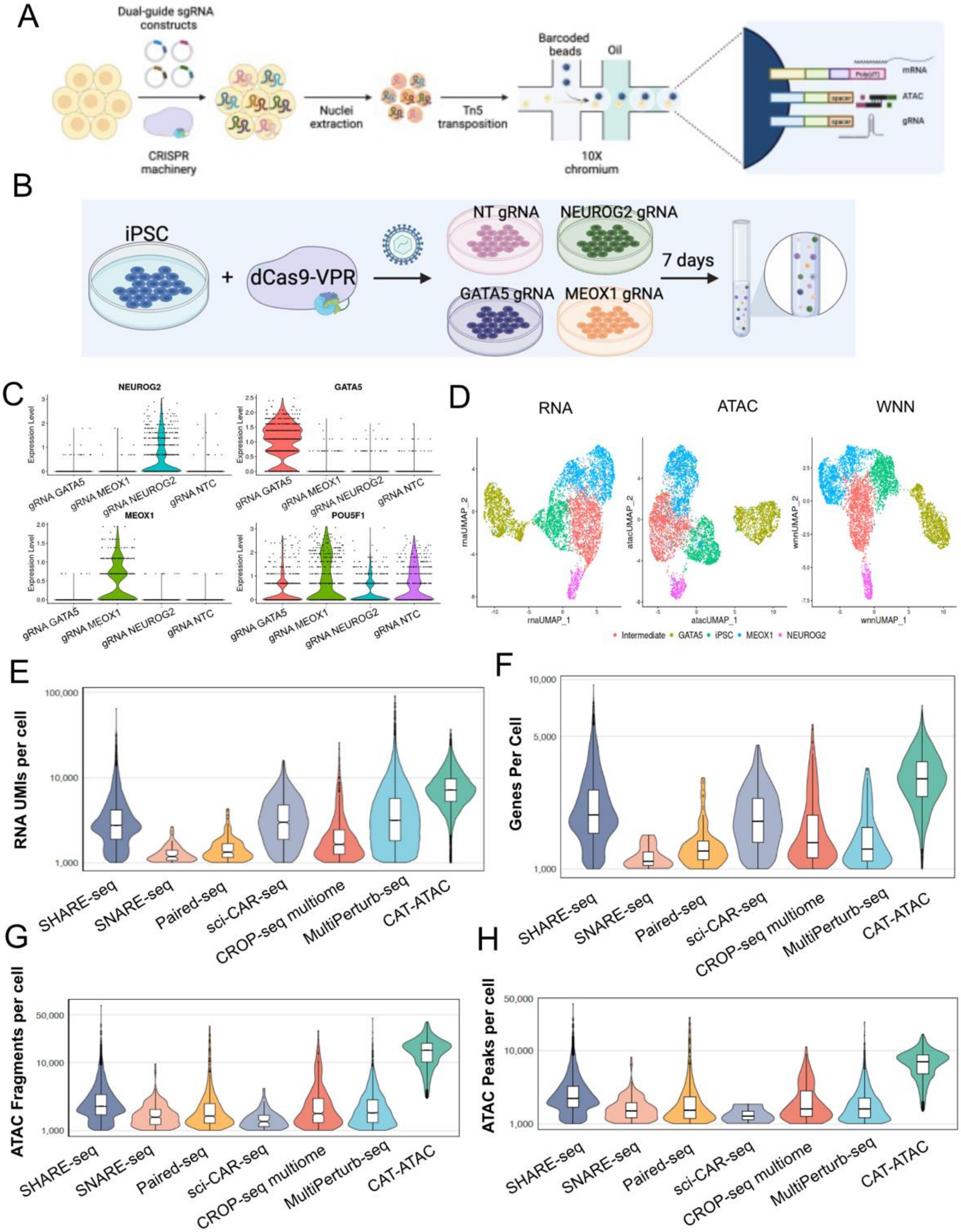

Supplementary Figure 1: CAT-ATAC QC and comparisons with other technologies, related to Figure 1:

A. Illustration of CAT-ATAC workflow
B. Pilot experiment overview
C. Violin plots showing protospacer expression in clusters shown in UMAP in Figure 1 panel F.
D. UMAPs based on RNA, ATAC and joint profiles for CAT-ATAC in iPSCs with 3 TFs (GATA5, NEUROG2, MEOX1) and 1 non-targeting control overexpressed by CRISPRa.
E. Violin plots comparing RNA UMIs per cell metric for various Multiome approaches with CAT-ATAC.
F. Violin plots comparing Genes per cell metric for various Multiome approaches with CAT-ATAC.
G. Violin plots comparing ATAC fragments per cell metric for various Multiome approaches with CAT-ATAC.
H. Violin plots comparing ATAC peaks per cell metric for various Multiome approaches with CAT-ATAC.

Supplementary Figure 2

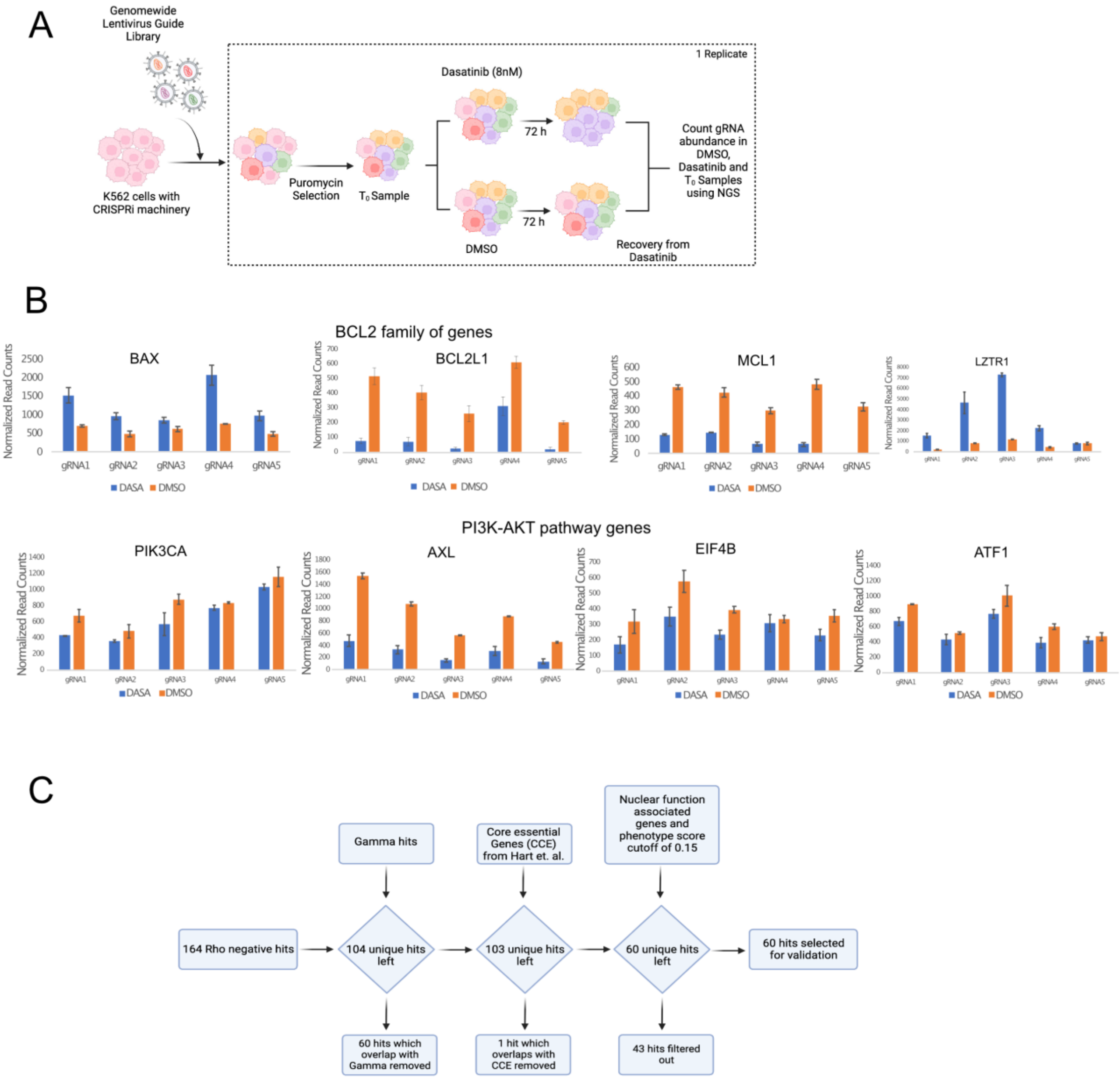

Supplementary Figure 2: Dasatinib genomewide screen design and QC, related to Figure 2:

A. Design of the dasatinib survival screen.
B. Normalized read counts of guides targeting some of the genes that were identified as hits and were highlighted in Figure 2 panel B. Error bars represent standard deviation.
C. Strategy to filter the sensitizing hits.

Supplementary Figure 3

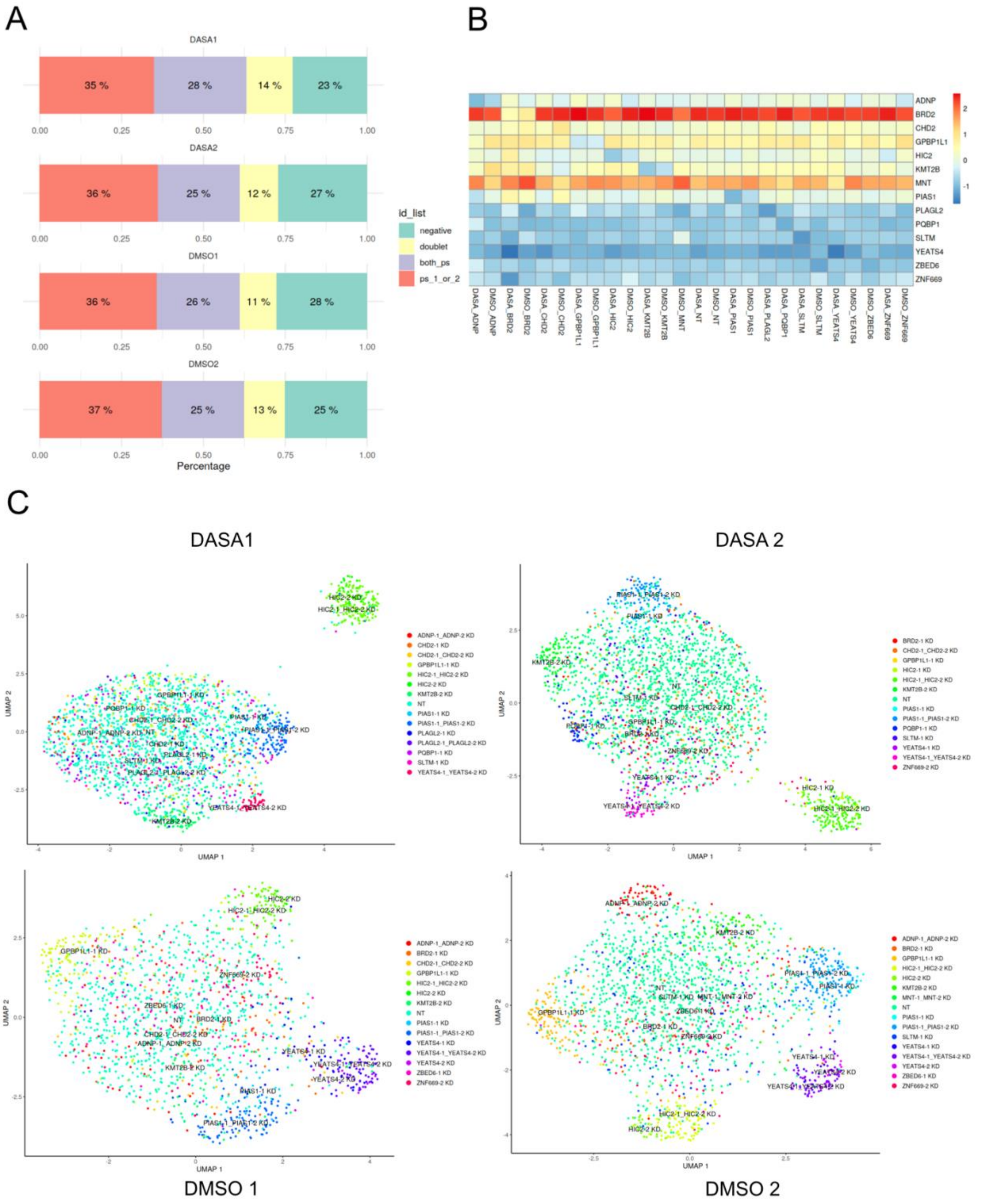

Supplementary Figure 3: CAT-ATAC QC for dasatinib screen, related to Figure 3:

A. Bar graph showing guide assignment for each of the 4 samples from the 10x Multiome screen.
B. Heatmap showing average accessibility at the promoters of targeted genes in both dasatinib and dmso treated samples.
C. UMAPs showing RNA expression profiles of cells for individual samples grouped by perturbation classes based on gRNA expression.

Supplementary Figure 4

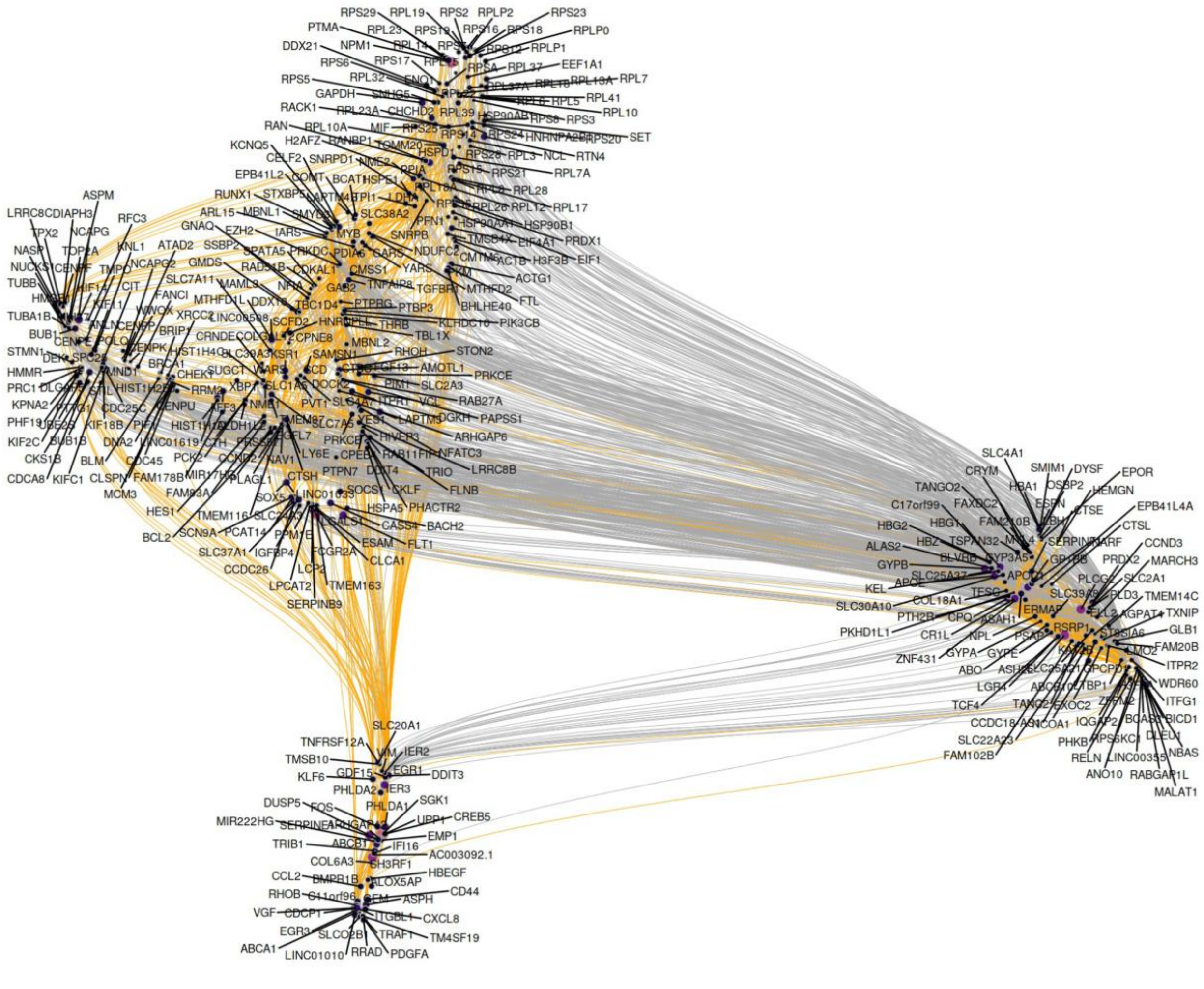

Supplementary Figure 4: Global GRN, related to figure 4:
Global GRN constructed using Pando

Supplementary Figure 5

A

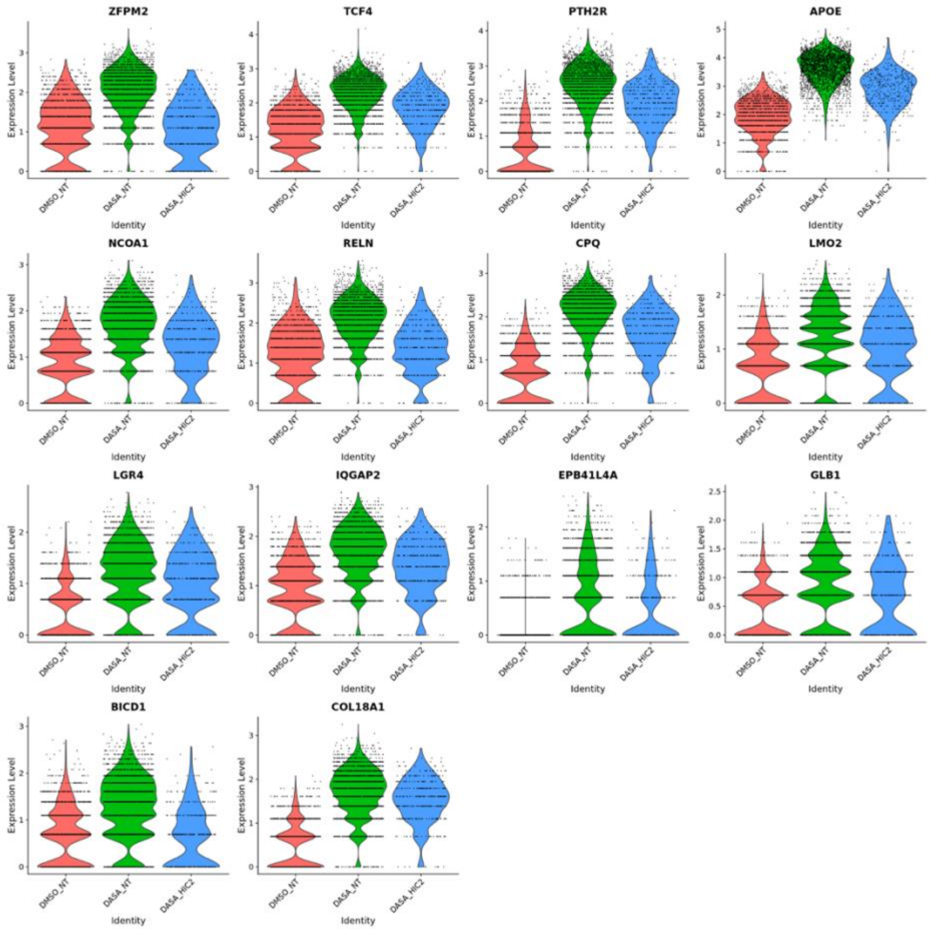

B

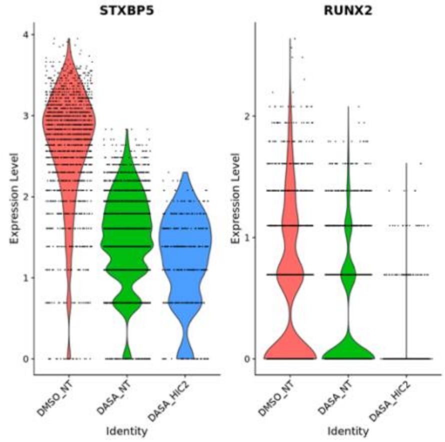

Supplementary Figure 5: Expression of genes in the subsetted GRN, related to figure 4:

A. Violin plots showing expression of genes having activating connections in the subsetted gene regulatory network.
B. Violin plots showing expression of genes having repressive connections in the subsetted gene regulatory network.

Supplementary Figure 6

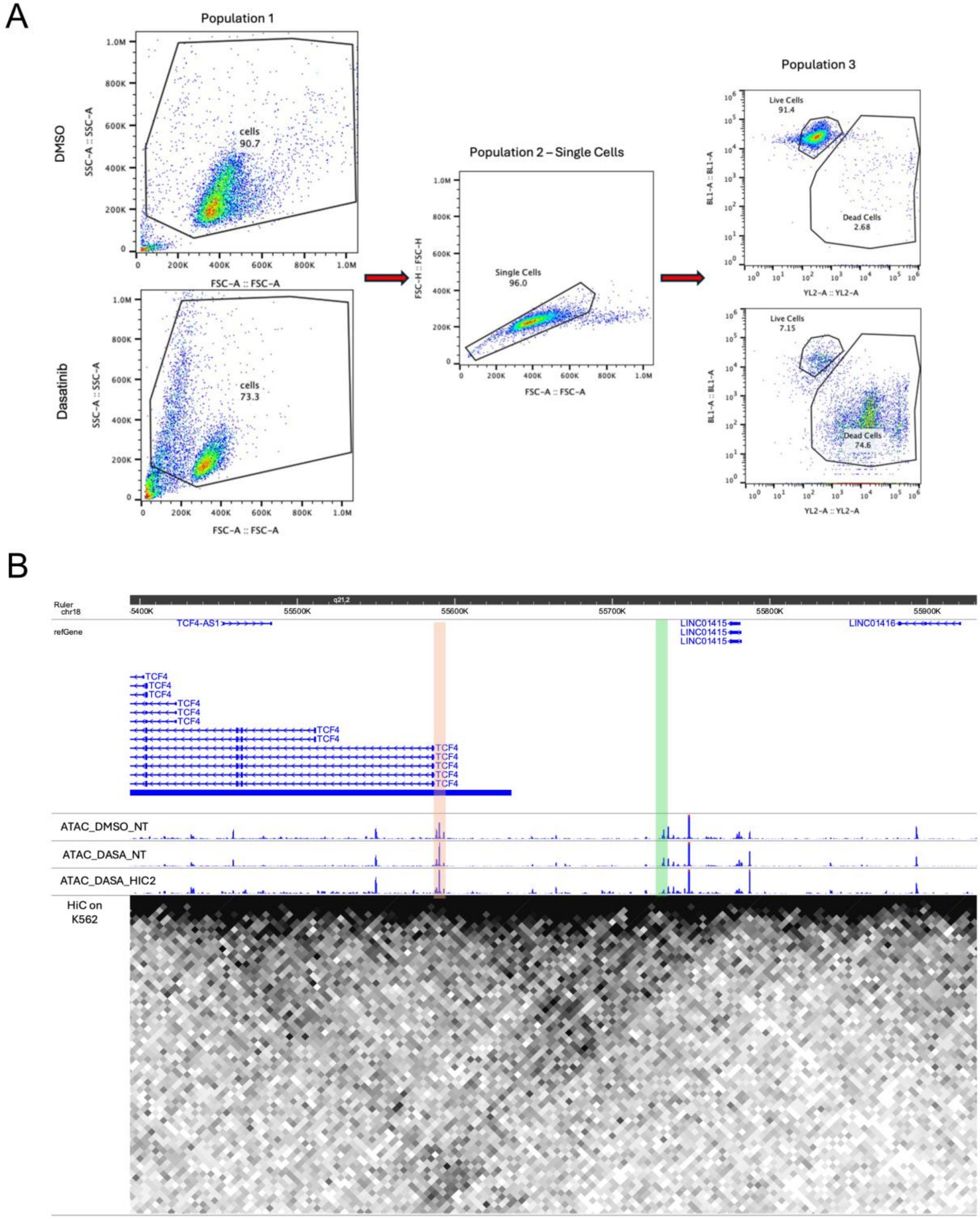

Supplementary Figure 6: Flow cytometry gating strategy and HiC data for TCF4 locus, related to Figure 4:

A. Gating strategy for flow cytometry data of k562/CRISPRi cells to study the effect of dasatinib on CRISPRi constructs targeted against HIC-2 and ZFPM2 stained with 1ug/ml EthD-1 and 2 μM calcein-AM analyzed by flow cytometry.
   The first panel shows all events with a gate (population 1) for cells. The second panel shows population 1 with a gate for singlets (population 2). The third panel shows Quarter 1 = Green or BL1-A for live cells and Quarter 3 = Red or YL2-A for dead cells (population 3).
B. Genome browser tracks showing the TCF4 locus. The orange and green highlighted areas mark the promoter and putative upstream enhancer. The last track shows HiC contacts in K562 cells at this locus from the 4D Nucleome datasets. We can see that the TCF4 promoter and the enhancer lies in a contact domain with relatively high contact frequency.

Supplementary Figure 7

A

A data.table: 1 × 8

pathway	pval	padj	log2err	ES	NES	size	leadingEdge
<chr>	<dbl>	<dbl>	<dbl>	<dbl>	<dbl>	<int>	<list>
WNT_SIGNALING	0.01552209	0.01552209	0.3807304	0.3862391	1.574156	46	FZD7, TL....

B

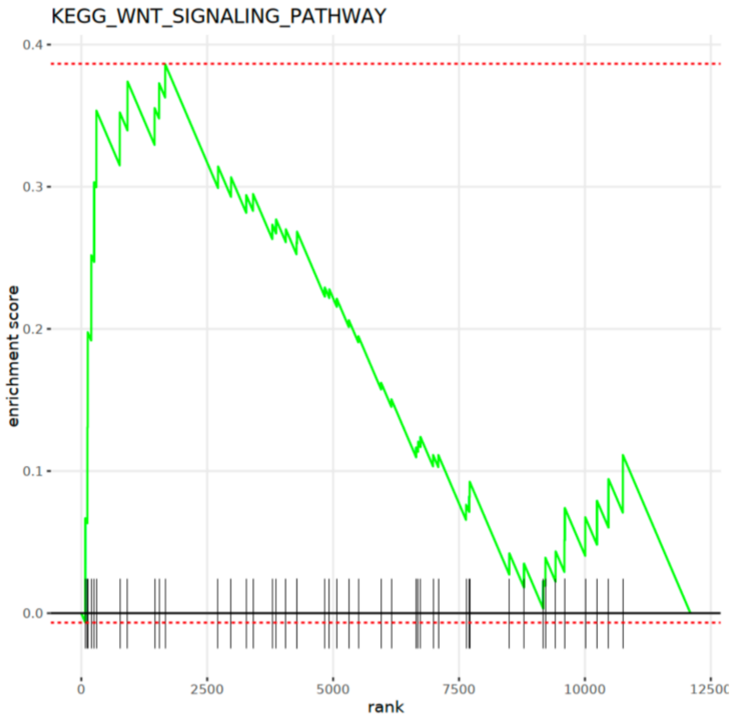

Supplementary Figure 7: GSEA for WNT pathway genes, related to Figure 5:

A. Table showing p-values for the GSEA performed using the KEGG_WNT_SIGNALING_PATHWAY gene set from the MSigDb database.
B. Enrichment plot showing enrichment of genes in the KEGG_WNT_SIGNALING_PATHWAY gene set in DASA_NT cells as compared to DASA_HIC2 cells.

Supplemental Methods S1, detailed CAT-ATAC protocol, related to STAR Methods

Before starting:

- Set heat block to 95°C for incubation of digitonin
- Cool swing-bucket centrifuge to 4°C

1. Anneal oligos 1 and 2 to make the RT splint duplex according to Oligo Annealing protocol by IDT. Store in -20°C.
2. Extract nuclei according to 10x protocol for cell lines.
3. Transpose nuclei according to 10x multiome protocol.
4. At **GEM Generation & Barcoding step (2.1)**, add spike-in RT primer (diluted to 0.5uM) and Hi-T4 ligase (NEB, M2622S).

	For 1x (ul)
Barcoding reagent mix	49.5
Template switch oligo	1.1
Reducing agent	1.9
RT-splint duplex, 0.5uM	1
Barcoding enzyme mix	7.5
Hi-T4 DNA ligase	3
Total	64

5. Incubate GEMs with the following conditions:

GEM Incubation		
Step	Temperature	Time
1	25°C	00:30:00
2	53°C	00:45:00
3	25°C	00:30:00
6	4°C	Hold

6. Follow 10x protocol until **Post GEM Incubation Cleanup – SPRIselect step (3.2)**. Instead of 1.8x SPRI beads cleanup, use 3.2x SPRI beads (160ul) and 1.8x isopropanol (90ul) to ensure single-stranded cDNA from sgRNAs can be purified.
7. Make ATAC and GEX libraries as instructed in 10x protocol.
8. For CRISPR capture library:
   i. Take 40ul pre-amp product, perform 0.6x-1.0x bead selection to remove large cDNA fraction. Add 60ul EB to make total volume 100ul. First beads addition is 60ul, second beads addition 40ul. Elute in 30ul.
   ii. Perform Feature PCR using primers 8 and 5, 5ul template, in a 100ul reaction volume with Kapa HiFi HotStart ReadyMix (Roche, KK2601).

Feature PCR		
	For 1x (ul)	x2.2
HiFi Master Mix	50	110
10uM Forward primer (oligo 8)	4	8.8
10uM Reverse primer (oligo 5)	4	8.8
	58	
DNA	5	
Water	37	81.4
Total	100	

Feature PCR Cycling (10 cycles)		
Step	Temperature	Time
1	98°C	00:00:45
2	98°C	00:00:20
3	58°C	00:00:05
4	72°C	00:00:05<br>Go to step 2, repeat 9x
5	72°C	00:01:00
6	4°C	Hold

i. Use 2ul to run on Tapestation D1000 HS. Check if amplicon size is within expected range.

ii. Beads cleanup with 0.8x-0.9x SPRI. Elute in 30ul. Confirm size of purified product with Tapestation D1000 HS again. Example TS trace is shown below.

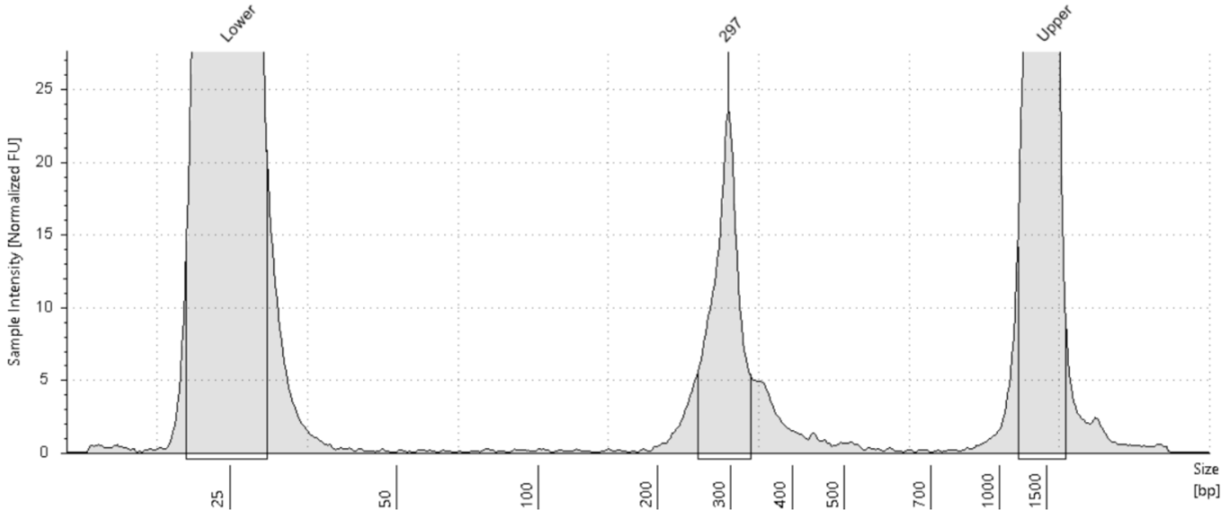

iii. Perform Sample Index PCR with primers 8 and 6a-f, 5ul template, in a 100ul reaction volume.

Sample Index PCR		
	For 1x (ul)	x2.2
HiFi Master Mix	50	110
10uM Forward primer (oligo 8)	4	8.8
10uM Reverse primer (oligo 6a-f)	4	8.8
	58	
DNA	5	
Water	37	81.4
Total	100	

Sample Index PCR Cycling (9 cycles)		
Step	Temperature	Time
1	98°C	00:00:45
2	98°C	00:00:20
3	60°C	00:00:30
4	72°C	00:00:20<br>Go to step 2, repeat 8x
5	72°C	00:01:00
6	4°C	Hold

i. Use 1ul to run on Tapestation D1000.
ii. Clean up with 0.7x-0.8x beads for double-sided size selection. Elute in 30ul. Use 1ul to run on Tapestation D1000 to confirm size. Example TS trace is shown below.

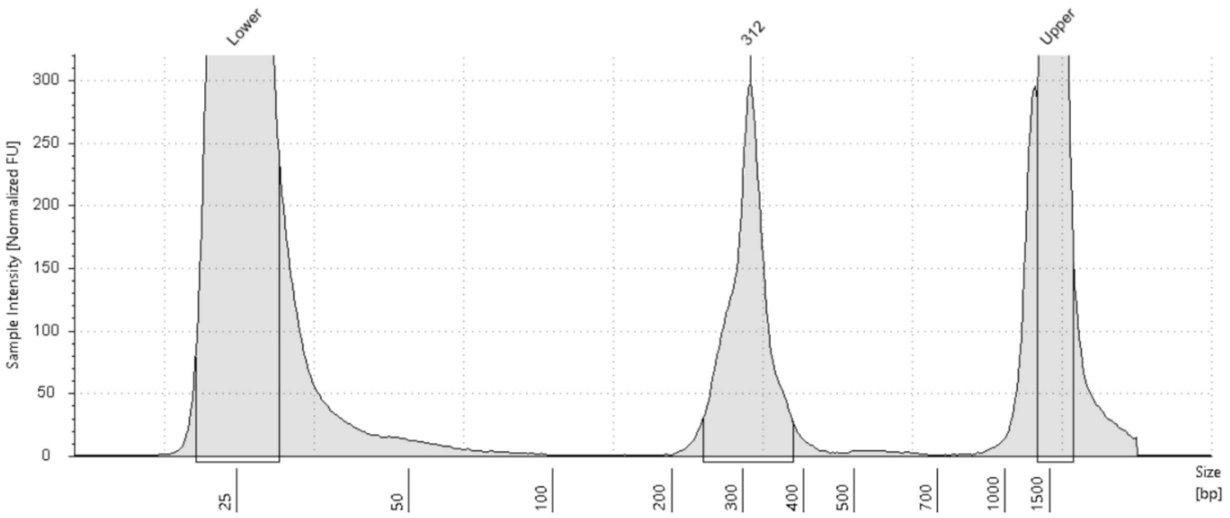

Further notes on CRISPR library construction and oligo sequences:

1. Structure of the CRISPR library is shown below:

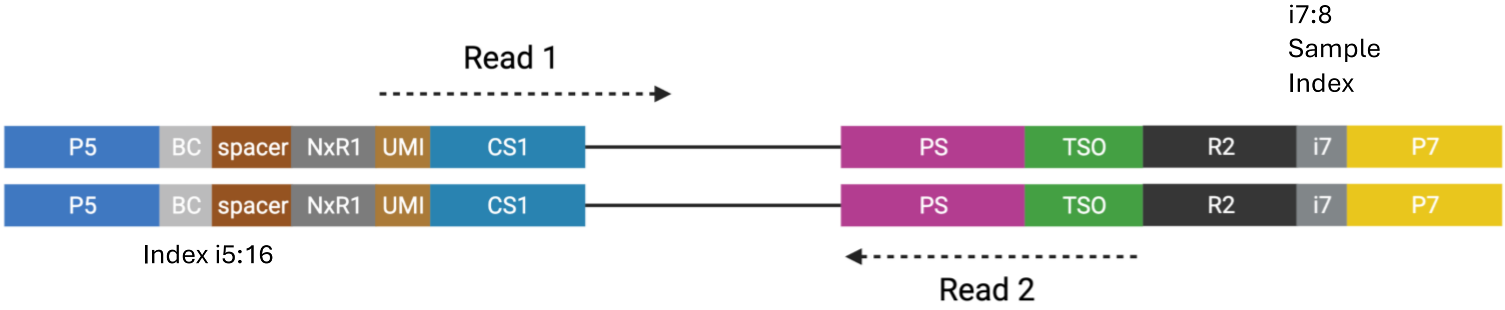

2. CRISPR library should be pooled with ATAC library and sequenced together. Sequencing configuration should be 100x8x24x100 (at least 75bp PE to be able to sequence through protospacer region). Aim for 25,000 read pairs per nucleus for ATAC library, and 5,000 read pairs per nucleus for CRISPR library.

3. Oligonucleotide sequences

Name	Description	Sequence
1	RT primer CS1_12bp UMI	/5Phos/TCGTCGGCAGCGTCAGATGTGTATAAGAGACAGNNNNNNNNNNNNTTGCTAGGACCGGCCTTAAAGC
2	splint with LNA	TGACGCTGCC+G+A+C+G+ACAGACGCG/3Phos/
5	R2-TSO primer	GTGACTGGAGTTCAGACGTGTGCTCTTCCGATCTAAGCAGTGGTATCAACGCAGAG
8	Partial P5 forward	AATGATACGGCGACCACCGAGA
6a	P7-i7-R2 primer A1	caagcagaagacggcatacgagatTTCGCAGTgtgactggagttcagacgtgtgctcttccgatct
6b	P7-i7-R2 primer A2	caagcagaagacggcatacgagatCGAGACTAgtgactggagttcagacgtgtgctcttccgatct
6c	P7-i7-R2 primer A3	caagcagaagacggcatacgagatACAGCTCAgtgactggagttcagacgtgtgctcttccgatct
6d	P7-i7-R2 primer B1	caagcagaagacggcatacgagatAAGTGTCGgtgactggagttcagacgtgtgctcttccgatct
6e	P7-i7-R2 primer A1_1	caagcagaagacggcatacgagatAGTAAACCgtgactggagttcagacgtgtgctcttccgatct
6f	P7-i7-R2 primer A1_2	caagcagaagacggcatacgagatCCGTTTAGgtgactggagttcagacgtgtgctcttccgatct

4. CRISPR library index sequences

Primer name	i7 index	i7 for sample sheet
6a	TTCGCAGT	ACTGCGAA
6b	CGAGACTA	TAGTCTCG
6c	ACAGCTCA	TGAGCTGT
6d	AAGTGTCG	CGACACTT
6e	AGTAAACC	GGTTTACT
6f	CCGTTTAG	CTAAACGG

5. Note: Do not use the following ATAC indexes from the multiome kit to avoid index clashing with CRISPR library indexes (hamming distance <2):

SI-NA-B1
SI-NA-A5
SI-NA-B6
SI-NA-G11
SI-NA-C11
